# PGRN acts as a novel regulator of mitochondrial homeostasis by facilitating mitophagy and mitochondrial biogenesis to prevent podocyte injury in diabetic nephropathy

**DOI:** 10.1038/s41419-019-1754-3

**Published:** 2019-07-08

**Authors:** Di Zhou, Meng Zhou, Ziying Wang, Yi Fu, Meng Jia, Xiaojie Wang, Min Liu, Yan Zhang, Yu Sun, Yi Lu, Wei Tang, Fan Yi

**Affiliations:** 1 The Key Laboratory of Infection and Immunity of Shandong Province, Departments of Pharmacology, School of Basic Medical Sciences, Shandong University, Jinan, China; 2 Departments of Biochemistry and Molecular Biology, School of Basic Medical Sciences, Shandong University, Jinan, China; 3 Departments of Pathogenic Biology, School of Basic Medical Sciences, Shandong University, Jinan, China; 4 The State Key Laboratory of Microbial Technology, Shandong University, Jinan, China

**Keywords:** Mitophagy, Mitochondria, Diabetic nephropathy

## Abstract

Mitochondrial dysfunction is considered as a key mediator in the pathogenesis of diabetic nephropathy (DN). Therapeutic strategies targeting mitochondrial dysfunction hold considerable promise for the treatment of DN. In this study, we investigated the role of progranulin (PGRN), a secreted glycoprotein, in mediating mitochondrial homeostasis and its therapeutic potential in DN. We found that the level of PGRN was significantly reduced in the kidney from STZ-induced diabetic mice and patients with biopsy-proven DN compared with healthy controls. In DN model, PGRN-deficient mice aggravated podocyte injury and proteinuria versus wild-type mice. Functionally, PGRN deficiency exacerbated mitochondrial damage and dysfunction in podocytes from diabetic mice. In vitro, treatment with recombinant human PGRN (rPGRN) attenuated high glucose-induced mitochondrial dysfunction in podocytes accompanied by enhanced mitochondrial biogenesis and mitophagy. Inhibition of mitophagy disturbed the protective effects of PGRN in high glucose-induced podocytotoxicity. Mechanistically, we demonstrated that PGRN maintained mitochondrial homeostasis via PGRN-Sirt1-PGC-1α/FoxO1 signaling-mediated mitochondrial biogenesis and mitophagy. Finally, we provided direct evidence for therapeutic potential of PGRN in mice with DN. This study provides new insights into the novel role of PGRN in maintaining mitochondrial homeostasis, suggesting that PGRN may be an innovative therapeutic strategy for treating patients with DN.

## Introduction

Diabetic nephropathy (DN) is one of the major microvascular complications of diabetes mellitus and the most common cause of end stage renal disease. Emerging evidence has shown that podocyte injury is a major prognostic determinant in the development of DN. Podocytes are the highly specialized terminally differentiated glomerular epithelial cells and play an essential role in maintain glomerular filtration barrier. Studies from diabetic patients have revealed the reduced number of podocytes and the abnormalities in podocyte structure and function in the early progression of DN^1–3^. In diabetic condition, podocytes lose specific markers of differentiation, undergo foot process effacement and detachment, and reduce the capacity to maintain the glomerular filtration barrier, thereby resulting in proteinuria^1^. Thus, therapies aimed at preventing podocyte injury or promoting podocyte repair are potential strategies for treating patients with DN^4^.

Despite the pathogenesis of DN being complex, emerging evidence has shown that mitochondrial dysfunction takes the center stage in the development and progression of diabetes and its complications^5^. Less mitochondrial proteins are expressed in both tubules and glomeruli of human diabetic kidney^6^. In particular, podocytes are sensitive to mitochondrial dysfunction. Abnormalities of mitochondria in podocytes have been found in animal models of streptozotocin (STZ)-induced DN^7^, and high fat diet-induced glomerulopathy^8^, highlighting the importance of podocyte mitochondrial dysfunction in the pathogenesis of DN. Therefore, it is remarkably important to search for new therapeutic and preventive strategies aimed at invigorating podocyte mitochondrial function by exploiting key components for mitochondrial homeostasis^9^, which involves a network of cellular processes including mitochondrial fission, fusion, biogenesis, and mitophagy. Disruption of these processes results in mitochondrial dysfunction and organ damage. Aberrant mitochondrial morphology and mitochondrial dysfunction are found in high glucose (HG)-treated podocytes due to the decreased levels of mitophagy^10^, indicating that mitophagy may play an essential role in the maintaining mitochondrial homeostasis and podocyte function.

Progranulin (PGRN), a secreted glycoprotein, is abundantly expressed in a broad range of tissues and cell types with pleiotropic functions including embryogenesis, inflammation, wound repair, neurodegeneration, and lysosome function. The anti-inflammatory properties of PGRN highlight its potential for novel therapeutic approaches for inflammatory diseases^11,12^. In the kidney, our previous studies have found that PGRN negatively regulates NOD2-mediated immune responses in acute kidney injury^13^. Although recent studies have observed that serum PGRN is elevated among patients with low eGFR and urinary PGRN correlates with albuminuria in diabetic patients^14^, the expression patterns of PGRN in the kidney and the contribution of PGRN to the pathogenesis of DN remain unclear. In this study, we found that the expression of PGRN was markedly reduced in renal biopsies from patients with DN and in the kidney from diabetic mice. Importantly, we explored a novel role of PGRN in maintaining mitochondrial homeostasis via PGRN-Sirt1-PGC-1α/FoxO1 signaling-mediated mitochondrial biogenesis and mitophagy in podocytes, suggesting that targeting mitochondrial function and cellular bioenergetics upstream of cellular damage by PGRN may provide unexpected opportunities for the treatment of DN.

## Materials and methods

### Human renal biopsy samples

Renal biopsies had been performed as part of routine clinical diagnostic investigation and the samples of renal biopsies were obtained from Department of Pathology, Shandong University School of Medicine. Control samples were obtained from the healthy kidney poles of individuals who underwent tumor nephrectomies without diabetes or renal disease. Among them, diabetic patients without nephropathy (DM-NN) were selected from patients who underwent nephrectomy for solitary renal cell carcinoma and had a concomitant diagnosis of type 2 diabetes. Histologic examinations and biochemical analysis (urine albumin-to-creatinine ratio < 30 mg/g) revealed no features of DN or other renal disease except for the solitary renal cell carcinoma. The investigations were conducted in accordance with the principles of the Declaration of Helsinki and were approved by the Research Ethics Committee of Shandong University after informed consent was obtained from the patients.

### Animal studies

PGRN-deficient mice (B6(Cg)-*Grn*^*tm1.1Aidi*^/J; *Grn*^*−/−*^) were purchased from the Jackson Laboratory (Bar Harbor, ME). Mouse models of diabetes were developed by injection of STZ into 8-week-old male *Grn*^*−/−*^ and age-matched C57BL/6 wild-type (WT) mice. Because mice with a C57BL/6 background do not develop lesions of DN readily after the induction of diabetes by STZ, uninephrectomization was performed to hasten the development of DN following previous studies^15,16^. Mice with fasting blood glucose (fasting for 8 h before the test) levels >15.0 mmol/l were considered as diabetic. All mice had unrestricted access to food and water and were maintained for 12 weeks in accordance with the Institutional Animal Care and Use Committee procedures of Shandong University. The physical and biochemical parameters of experimental animals were shown in Supplementary Table 1. To implicate the role of PGRN in vivo, recombinant human PGRN (rPGRN, 10 mg/kg) was administrated to STZ-induced diabetic mice model by intraperitoneal injection 2 times/week for 6 weeks beginning at 6 weeks following STZ injection. All protocols were approved by the Institutional Animal Care and Use Committee of Shandong University (Document no. LL-201501025) and conducted in accordance with the National Institutes of Health Guide for the Care and Use of Laboratory Animals.

### Cell culture and treatments

Human podocytes, rat glomerular endothelial cells, and rat glomerular mesangial cells were cultured as described previously^16^. To study the effect of PGRN, rPGRN was added in the medium at a final concentration of 500 ng/ml.

### Histology examination

Periodic-acid-Schiff (PAS) staining was performed for the detection of structures of formalin-fixed kidney sections. A semiquantitative score of 0–4 was used to assess the mesangial matrix expansion in glomerular tuft area^17^.

### Gene silencing of target genes

Short interfering RNA (siRNA) was delivered into podocytes by the Lipofectamine 2000 reagent (Invitrogen, Carlsbad, CA) following the manufacturer’s protocol. siRNA against SIRT1 (sc-40986) and scramble control (sc-37007) were obtained from Santa Cruz Biotechnology (Santa Cruz, CA). siRNAs against PARK2 and their negative control siRNA were synthesized by RiboBio (Guangzhou, China). The sequences of siRNA oligonucleotides were as follows: human PARK2, 5′-GGATCAGCAGAGCATTGTTCA-3′; control siRNA, 5′-UUCUCCGAACGUGUCACGU -3′^18^.

### Flow cytometry

Podocyte apoptosis was determined by fluorescein isothiocyanate (FITC)-conjugated Annexin V and propidium iodide (PI) staining as described^13^.

### Real time RT-PCR

The mRNA levels for target genes were analyzed by real-time quantitative RT-PCR using a Bio-Rad iCycler system (Bio-Rad, Hercules, CA). The specific primers for target genes were listed in Supplementary Table 2.

### TUNEL assay

Cell death in the kidney was detected by TUNEL assay following the manufacturer’s protocols (Roche Diagnostics, Mannheim, Germany).

### Transmission electron microscopy (TEM)

Electron microscopic sample handling and detection were performed by the electron microscopic core lab of Shandong University as described^16,19^. Five glomeruli were randomly selected from each mouse and 10 electron micrographs were taken in each glomerulus. TEM images of kidney tissues and cultured podocytes were analyzed using NIH ImageJ software (National Institutes of Health, NIH, Bethesda, MD).

### Western blot analysis

Western blot analysis was performed as described previously^20^. Antibodies used for western blot analysis were summarized in Supplementary Table 3. The uncropped blots were shown in Supplementary Fig. 4.

### Immunoprecipitation (IP)

Acetyl-lysine antibody (2 µg) was incubated with 30 µl Protein A&G magnetic beads (Selleck, Huston, TX) for one hour at room temperature with constant rotation. The magnetic beads and the 500 µg sample lysates were incubated overnight at 4 °C. After elution, the immunoprecipitated proteins were detected by western blot using PGC-1α antibody.

### Immunohistochemical staining

Immunohistochemical staining was performed as described^12^. Semiquantitation of immunohistochemical staining was performed as described^21^.

### Immunofluorescence staining and confocal microscopy

Immunofluorescent staining was performed as described^22,23^, and fluorescent images were obtained with a LSM780 laser scanning confocal microscope (ZEISS, Germany) equipped with a Plan-Apochromat 63×/1.4 objective. Antibodies used for immunofluorescence staining were summarized in Supplementary Table 3. Mitochondria in podocytes were visualized with 100 nM MitoTracker Green (Beyotime, China) applied for 30 min at 37 °C before fixation. Mitochondrial length was quantified by using NIH ImageJ software.

### Mitochondrial membrane potential (MMP) assay

MMP was detected by the mitochondrial membrane potential assay kit with JC-1 following the manufacturer’s protocol (Beyotime, China).

### Quantification of mitochondrial DNA content

The relative copy number of mitochondrial DNA was detected by real-time quantitative PCR assay, which was performed on a Bio-Rad iCycler system (Bio-Rad, Hercules, CA) using SYBR Green methods. Total cellular DNA was extracted using the Genomic DNA Preparation Kit (Beyotime, China) following the manufacturer’s protocol. DNA primers were designed to detect Cyt b and cytochrome c oxidase subunit II (COII), as markers for mitochondrial DNA (mtDNA), and β-actin, as a marker for nuclear DNA (nDNA) (the sequence specific primers used in this study were listed in Supplementary Table 2). In brief, 1 μg DNA was amplified in a 20 μl volume containing 10 μl 2× SYBR Green PCR Mix and 0.5 μM of each primer. Real-time quantitative PCR conditions were 95 °C for 3 min and 39 cycles of 95 °C for 10 s, followed by 60 °C for 30 s. The relative copy number of mitochondrial DNA was represented by the ratio of mitochondrial to nuclear DNA (mtDNA:nDNA), which was calculated by the 2^*−*ΔΔCT^ method.

### Preparation of rPGRN protein

Generation of PGRN stable line and purification of rPGRN was described previously^12,13^.

### Statistical analyses

Data are expressed as means ± SEM. The significance of the differences in mean values between and within multiple groups was examined by one-way ANOVA followed by Duncan’s multiple range tests. *P* < 0.05 was considered statistically significant.

## Results

### The levels of PGRN were reduced in renal biopsies from patients with DN and in the kidney from diabetic mice

**Fig. 1:**
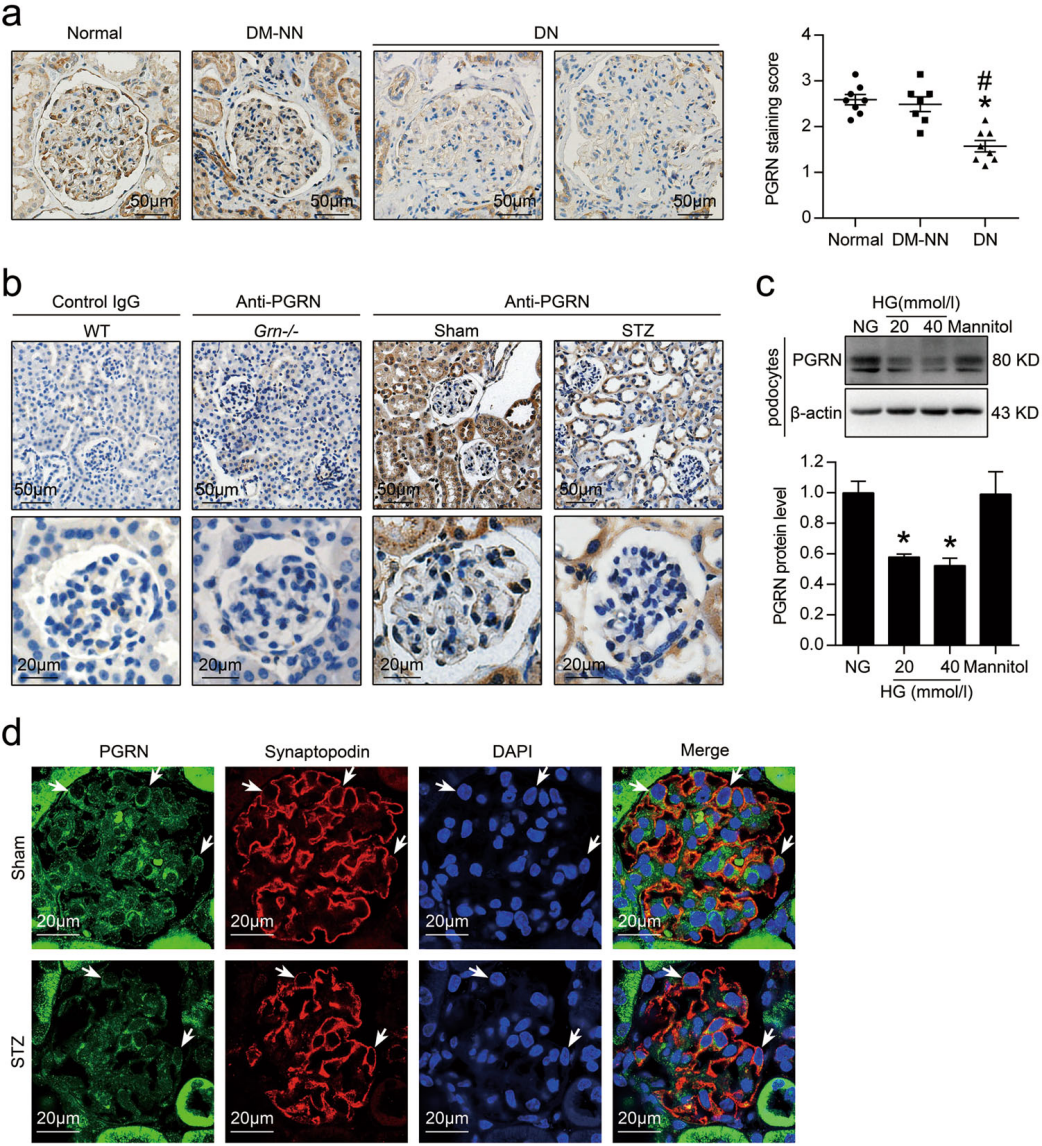
The level of PGRN was significantly reduced in the kidney under diabetic conditions. **a** Representative photomicrographs and semiquantitation of PGRN immunohistochemical staining in human renal cortical tissue from normal subjects (*n* = 8), patients with diabetic nephropathy (DN) (*n* = 8), or diabetic patients without nephropathy (DM-NN) (*n* = 7). *P < 0.05 vs. Normal, ^#^*P* < 0.05 vs. DM-NN. **b** Representative photomicrographs of PGRN immunohistochemical staining in the kidney from wild-type (WT) and PGRN-deficient (*Grn*^*−/−*^) mice (*n* = 6). Negative control by omission of the corresponding primary antibodies demonstrated no nonspecific staining. **c** Representative western blot gel documents and summarized data showing the relative protein levels of PGRN in human podocytes treated with high glucose (HG, final concentration 20 or 40 mmol/l in medium) for 48 h. *P < 0.05 vs. NG treatment. **d** Representative confocal microscopic images showing the expression of PGRN in podocytes of kidney from STZ-induced diabetic mice (*n* = 6), synaptopodin was used as a podocytes marker. The arrows indicate representative podocytes

As shown in Fig. 1a, immunohistochemical (IHC) staining showed that the levels of PGRN were reduced in renal biopsies from DN (*n* = 8) compared with normal subjects (*n* = 8) or diabetic patients without nephropathy (DM-NN, *n* = 7). The reduction of PGRN was also revealed in the kidney from STZ-induced diabetic mice by IHC staining as compared with sham-operated mice (Fig. 1b). Meanwhile, no staining with anti-PGRN in the kidney from PGRN-deficient mice (*Grn*^*−/−*^) was observed, indicating the specificity of the PGRN immunostaining. Considering that podocytes, endothelial cells, and mesangial cells are the major cell components in glomeruli, we then detected the expression pattern of PGRN in these cells with HG treatment. HG treatment significantly decreased the expression of PGRN in podocytes (Fig. 1c), which was further confirmed in vivo, by using double immunostaining for PGRN (green) and podocyte-specific marker (red), synaptopodin (Fig. 1d). Although there was a decrease tendency in glomerular endothelial cells (GECs), HG treatment had no significant effect on the expression of PGRN in glomerular endothelial cells and mesangial cells (Supplementary Fig. 1), indicating the cell-specific expression pattern of PGRN.

### PGRN deficiency exacerbated renal injury in diabetic mice

**Fig. 2:**
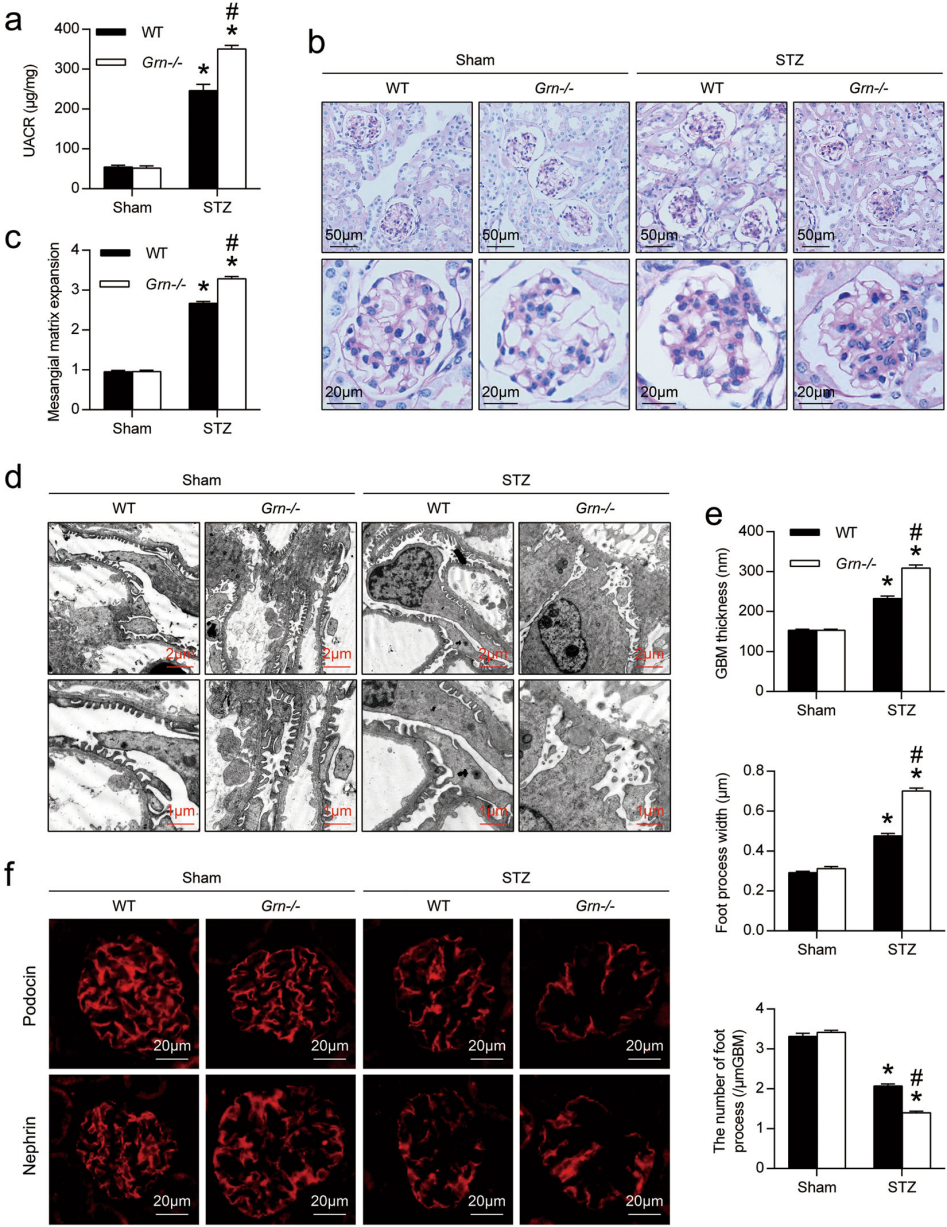
PGRN deficiency exacerbated podocyte injury and proteinuria in DN. **a** Urine albumin-to-creatinine ratio (UACR) in different groups of mice. **b** Representative images of periodic-acid-Schiff (PAS) staining of kidney sections in different groups of mice. **c** Quantification of Mesangial Matrix Index of glomerulus from different groups of mice. **d** Representative transmission electron microscopy (TEM) images showing morphological changes in the podocyte foot process in different groups of mice. **e** Quantitative assessment of glomerular filtration barrier integrity, including glomerular basement membrane (GBM) thickness, foot process width and the number of foot processes/μm GBM. *P < 0.05 vs. sham-operated mice, ^#^*P* < 0.05 vs. WT diabetic mice (*n* = 6). **f** Representative microscopic images showing the expressions of podocin and nephrin in the kidney from different groups of mice

As shown in Fig. 2a, compared with WT diabetic mice, the levels of urinary albumin excretion were increased in PGRN-deficient (*Grn*^*−/−*^) diabetic mice. *Grn*^*−/−*^ diabetic mice developed more severe morphological injuries as evidenced by increased mesangial expansion, hypercellularity and capillary collapse in the glomerulus (Fig. 2b, c). Transmission electron microscopy (TEM) further revealed that PGRN deficiency exacerbated podocyte injuries in diabetic mice, as demonstrated by loss of foot processes along the glomerular basement membrane (GBM), podocyte foot process broadening and effacement (Fig. 2d, e). In parallel with histological changes of glomeruli, the slit diaphragm proteins podocin and nephrin along the glomerular capillary loop in *Grn*^*−/−*^ diabetic mice were further reduced (Fig. 2f).

### PGRN deficiency exacerbated cell death and mitochondrial damage in podocytes from diabetic mice

**Fig. 3:**
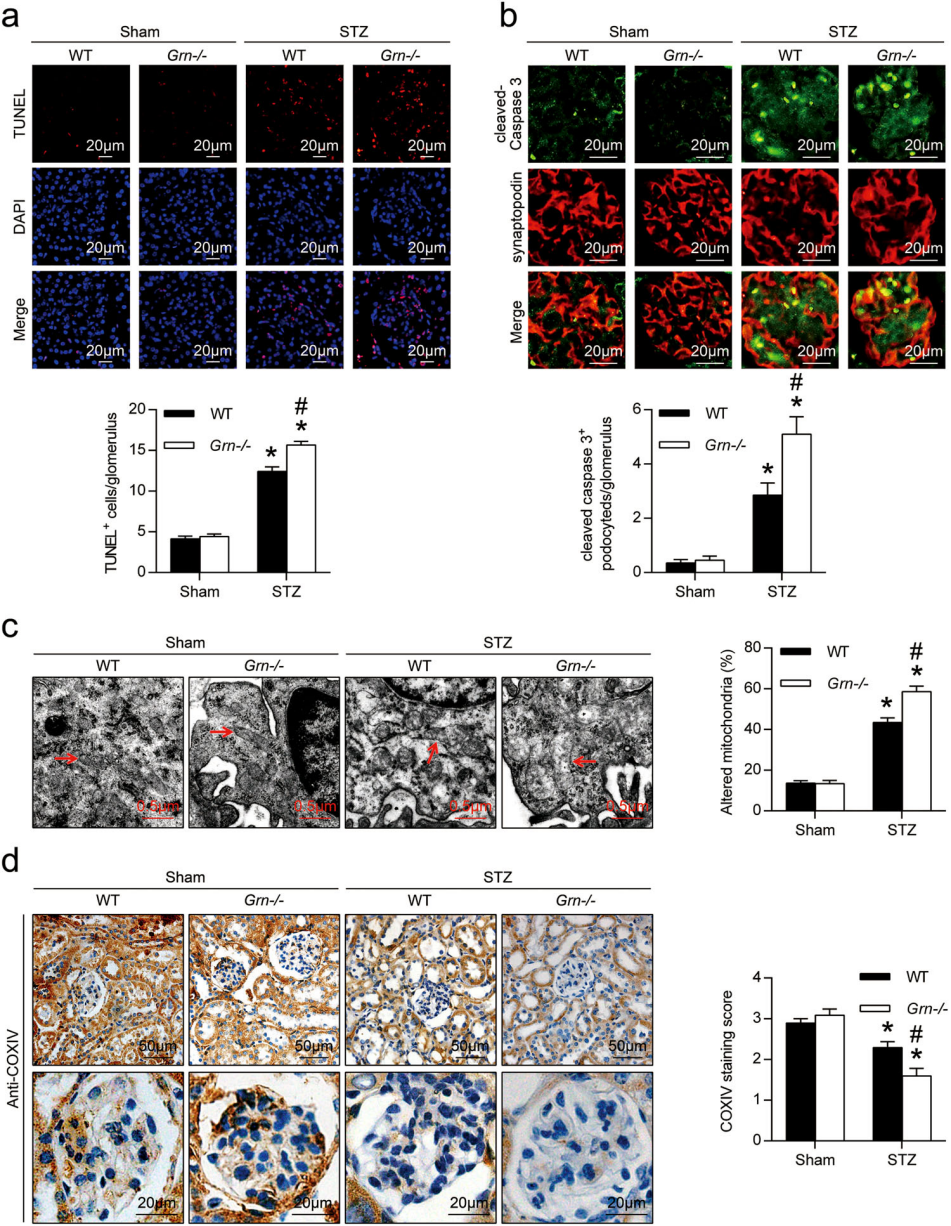
The cell death and mitochondrial damage were further exacerbated in podocytes of Grn^−/−^ diabetic mice. **a** In situ terminal deoxynucleotidyl transferase-mediated UTP nick-end labeling (TUNEL) assays were performed to assess the cell death in glomerulus. Nuclei were revealed using 4′,6-diamidino-2-phenylindole (DAPI) staining. Quantitative assessment of the number of cell death (number of TUNEL-positive cells per glomerulus). **b** Double immunofluorescence staining for cleaved-caspase 3 (green) and synaptopodin (red) in the kidney from different groups of mice. Quantitative assessment of the number of cleaved-caspase 3-positive podocytes in glomerulus. **c** Representative TEM images of glomeruli demonstrating mitochondrial morphology in podocytes of kidney sections and quantification of percentage of altered mitochondria characterized by mitochondria swelling, vacuolization and cristae fragmentation in podocytes from different groups of mice. Red arrows indicate representative mitochondria. **d** Representative photomicrographs and semiquantitation of cytochrome C oxidase (complex IV) subunit IV isoform 1 (COXIV) immunohistochemical staining in the kidney from different groups of mice. *P < 0.05 vs. sham-operated mice, ^#^*P* < 0.05 vs. WT diabetic mice (*n* = 6)

By TUNEL assay, we found that the number of apoptotic cells was significantly increased in glomeruli from WT diabetic mice compared with controls, which was further increased in *Grn*^*−/−*^ diabetic mice (Fig. 3a). To further identify podocyte apoptosis in glomeruli, double immunofluorescence staining with antibodies to synaptopodin and active fragments of caspase-3 was carried out. As shown in Fig. 3b, a very faint staining of active fragments of caspase-3 was observed in podocytes from WT and *Grn*^*−/−*^ control mice, while a strong staining in podocytes from diabetic mice. PGRN deficiency enhanced the number of cleaved-caspase 3-positive podocytes in glomeruli of diabetic mice (Fig. 3b). By TEM, we observed the mitochondrial damages in podocytes and glomerular endothelial cells from diabetic mice which were further exacerbated by PGRN deficiency as evidenced by changes of mitochondrial shape, size, and organization of cristae (Fig. 3c, Supplementary Fig. 2a). Moreover, PGRN deficiency further reduced the levels of cytochrome C oxidase (complex IV) subunit IV isoform 1 (COXIV) in glomeruli of diabetic mice (Fig. 3d).

### PGRN attenuated HG-induced mitochondrial damage and dysfunction in podocytes

**Fig. 4:**
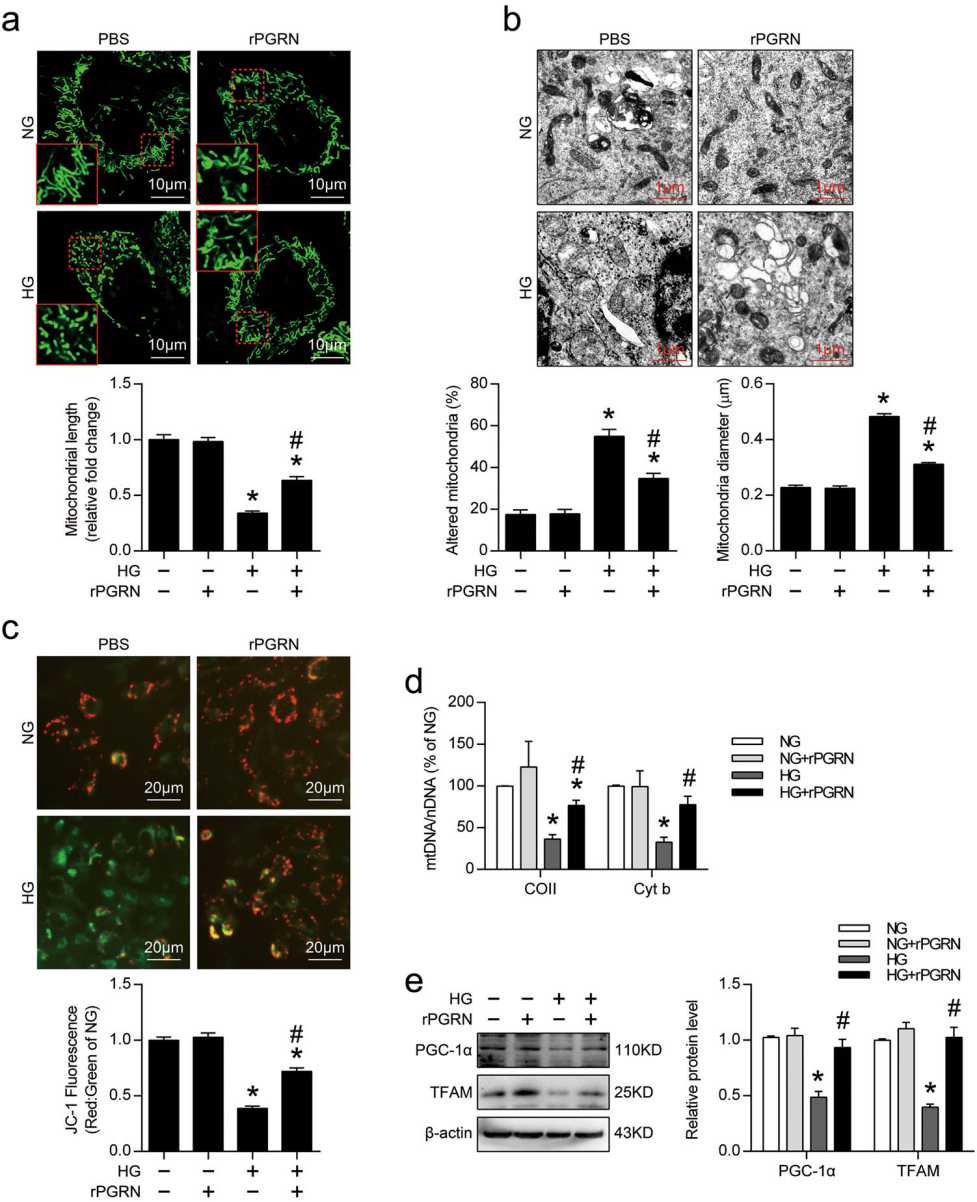
PGRN protected against HG-induced mitochondrial damage and dysfunction, and restored HG-reduced mitochondrial biogenesis in podocytes. **a** Representative images for MitoTracker green staining showing mitochondrial morphology and quantification of mitochondrial length in live cultured podocytes with different treatments. The results were normalized to the mitochondrial length of NG-treated podocytes. **b** Representative TEM images showing mitochondrial morphology and quantification of the percentage of altered mitochondria and mitochondria diameter of live cultured podocytes with different treatments. **c** Representative images of podocytes stained with JC-1 and quantification of JC-1 fluorescence (red-to-green ratio) showing changes in fluorescence intensity in podocytes with different treatments. JC-1 fluorescence was normalized with the red-to-green ratio of NG-treated podocytes. **d** Relative mitochondrial DNA content (mtDNA:nDNA) in podocytes with different treatments. The results were normalized to the ratio of mtDNA:nDNA of NG-treated podocytes. **e** Representative western blot gel documents and summarized data showing the expression levels of PGC-1α and TFAM in podocytes with different treatments. *P < 0.05 vs. NG treatment, ^#^*P* < 0.05 vs. HG treatment

In vitro, podocytes had higher percentages of fragmented mitochondria under HG condition, rPGRN treatment significantly inhibited HG-induced mitochondrial fission (Fig. 4a), and preserved HG-induced mitochondrial abnormalities by maintaining the mitochondrial morphology and diameter in podocytes (Fig. 4b). In addition, rPGRN effectively attenuated the HG-induced decline of MMP (Fig. 4c). Mitochondrial content was also assessed by mitochondrial DNA copy determination. We found that rPGRN treatment attenuated the HG-induced decline of the ratio of mitochondrial to nuclear DNA (mtDNA:nDNA) in podocytes (Fig. 4d). At the molecular levels, HG reduced the protein level of PGC-1α, the master regulator of mitochondrial biogenesis, which was reversed by rPGRN. The protein level of mitochondrial transcription factor A (TFAM) that involved in mitochondrial DNA replication/translation was also enhanced by rPGRN in podocytes under HG condition (Fig. 4e). These data suggest that PGRN has a mitochondria-protective role by maintaining mitochondria biogenesis in podocytes under HG condition.

### PGRN restored HG-reduced mitophagy in podocytes

**Fig. 5:**
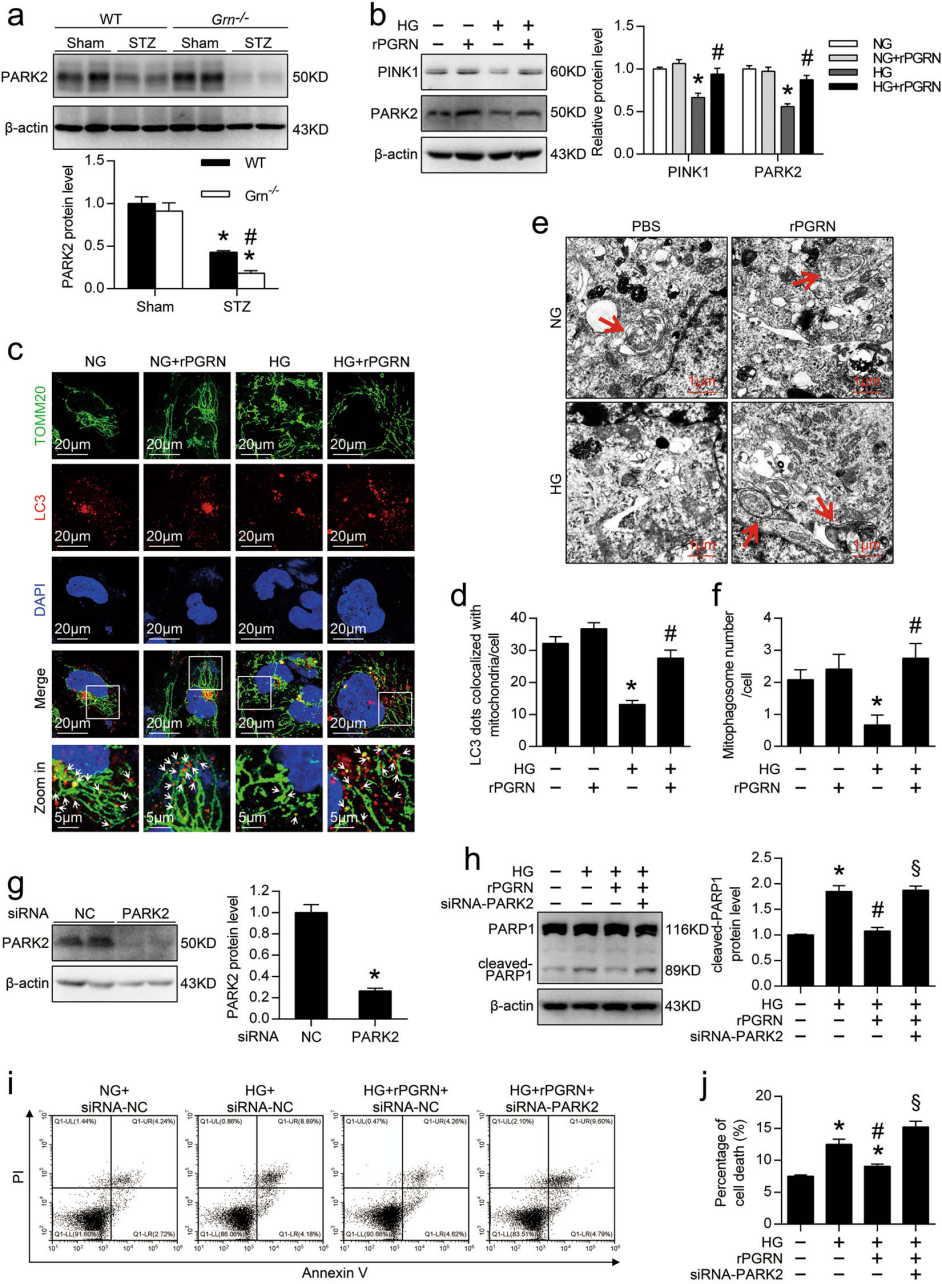
PGRN rescued HG-reduced mitophagy in podocytes. **a** Representative western blot gel documents and summarized data showing the protein levels of PARK2 in kidney from different groups of mice. *P < 0.05 vs. sham-operated mice, ^#^*P* < 0.05 vs. WT diabetic mice (*n* = 6). **b** Representative western blot gel documents and summarized data showing the relative protein levels of PINK1 and PARK2 in podocytes with different treatments. **c** Representative confocal microscopic images of double immunofluorescence staining for TOMM20 (green) and LC3 (red) in podocytes with different treatments. Nuclei were revealed using DAPI staining. White arrows indicate colocalization of autophagosomes (LC3) and mitochondria (TOMM20). **d** Quantitative analysis of mitophagosome formation represented by colocalization of autophagosomes (LC3) and mitochondria (TOMM20). **e** Representative TEM images demonstrating mitophagosomes (mitophagosomes engulfing mitochondria) in podocytes with different treatments. Red arrows indicate representative mitophagosomes. **f** Quantification of the numbers of mitophagic vacuoles of each group. *P < 0.05 vs. NG treatment, ^#^*P* < 0.05 vs. HG treatment. **g** Representative western blot gel documents and summarized data showing the relative protein level of PARK2 in siRNA-NC or siRNA-PARK2-transfected podocytes. *P < 0.05 vs. siRNA-NC. **h** Representative western blot gel documents and summarized data showing the expression levels of cleaved-PARP1 in podocytes with different treatments. **i** Representative flow cytometry analysis depicting the detection of apoptosis in podocytes with different treatments stained with fluorescein isothiocyanate (FITC)-conjugated Annexin V and propidium iodide (PI). **j** Quantitative data expressing overall percentage of cell death (annexin V-FITC-positive), including the amount of apoptotic and necrotic cells determined by flow cytometric analysis in podocytes with different treatments. *P < 0.05 vs. NG + siRNA-NC, ^#^*P* < 0.05 vs. HG + siRNA-NC, ^§^*P* < 0.05 vs. HG + rPGRN + siRNA-NC

In vivo, PGRN deficiency further decreased the levels of E3 ubiquitin-protein ligase Parkin (PARK2) in the kidney from diabetic mice (Fig. 5a). Consistently, in vitro, the live cultured podocytes showed remarkable decreases in PTEN-induced putative kinase 1 (PINK1) and PARK2 expression under HG condition, which was rescued by rPGRN treatment (Fig. 5b). By using double immunostaining for TOMM20 and LC3, markers for mitochondria and autophagosome individually, we found more colocalization of mitochondria and autophagosomes in rPGRN-treated podocytes under HG conditions, indicating that PGRN promotes the formation of mitophagogsomes (Fig. 5c, d). Consistently, TEM showed that rPGRN significantly increased the number of mitophagic vacuoles in podocytes under HG condition (Fig. 5e, f). Importantly, inhibition of PARK2 expression (Fig. 5g) disturbed the protective role of PGRN in HG-induced podocytotoxicity as evidenced by re-elevated the cleavage of PARP1 (Fig. 5h) and the percentage of cell death (Fig. 5i, j).

### PGRN induced the expression of Sirt1 and reduced the acetylation levels of PGC-1α and FoxO1 in HG-treated podocytes

**Fig. 6:**
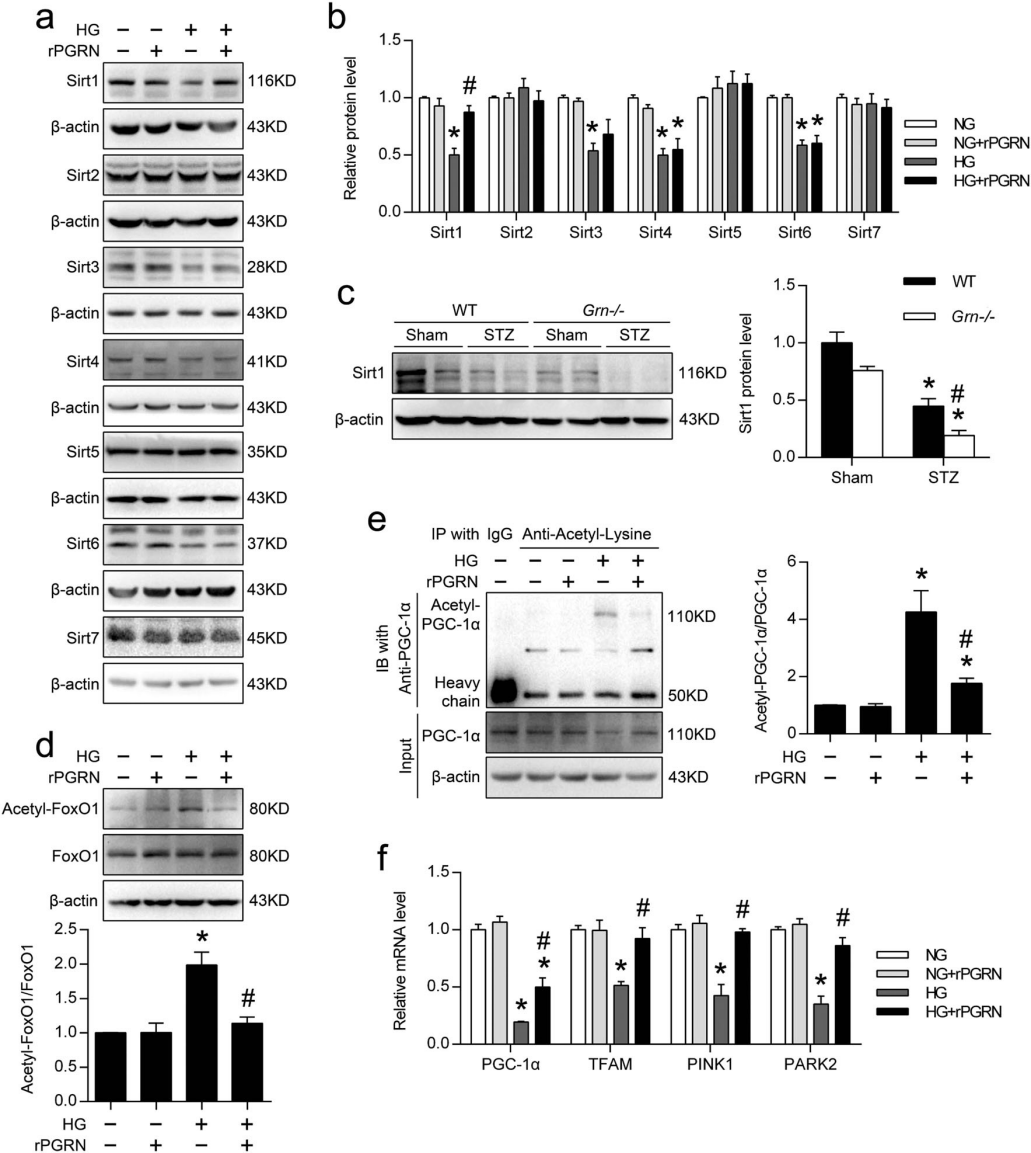
PGRN activated Sirt1-PGC-1α/FoxO1 pathway in HG-treated podocytes. **a** Representative western blot gel documents of Sirts in podocytes with different treatments. **b** Summarized data showing the expression levels of Sirts in podocytes with different treatments. *P < 0.05 vs. NG treatment, ^#^*P* < 0.05 vs. HG treatment. **c** Representative western blot gel documents and summarized data showing the expression levels of Sirt1 in kidney from different groups of mice. *P < 0.05 vs. sham-operated mice, ^#^*P* < 0.05 vs. WT diabetic mice (*n* = 6). **d** Representative western blot gel documents and summarized data showing the levels of acetylated-FoxO1 in podocytes with different treatments. **e** Representative western blot gel documents and summarized data showing the levels of acetylated-PGC-1α in podocytes with different treatments. The acetylated proteins were immunoprecipitated with an antibody to acetylated lysine and the presence of PGC-1α in the immune-complex was assessed by western blot with the antibody to PGC-1α. **f** Relative mRNA levels of PGC-1α, TFAM, PINK1, and PARK2 in podocytes with different treatments. *P < 0.05 vs. NG treatment, ^#^*P* < 0.05 vs. HG treatment

Considering that histone deacetylase Sirt1 is extensively implicated in both mitochondrial biogenesis and mitophagy, we then measured the levels of Sirt1 and other sirtuin members in podocytes in response to PGRN. We found that HG reduced the levels of Sirt1, Sirt3, Sirt4, and Sirt6 in podocytes. However, only the expression of Sirt1 was markedly restored by rPGRN treatment, although the level of Sirt3 had an increase tendency (Fig. 6a, b). In vivo, a more significant decrease of Sirt1 in glomeruli from diabetic *Grn*^*−/−*^ mice was observed as compared with diabetic WT mice (Fig. 6c). Subsequently, the acetylation levels of Sirt1 downstream targets including FoxO1, PGC-1α that are closely associated with the regulation of mitochondrial homeostasis, were significantly decreased in the presence of rPGRN (Fig. 6d, e). As a result, rPGRN significantly rescued the mRNA levels of PGC-1α, TFAM, PINK1, and PARK2 in podocytes with HG treatment (Fig. 6f).

### Inhibition of Sirt1 expression counteracted the protective effects of PGRN in podocytes with HG treatment

**Fig. 7:**
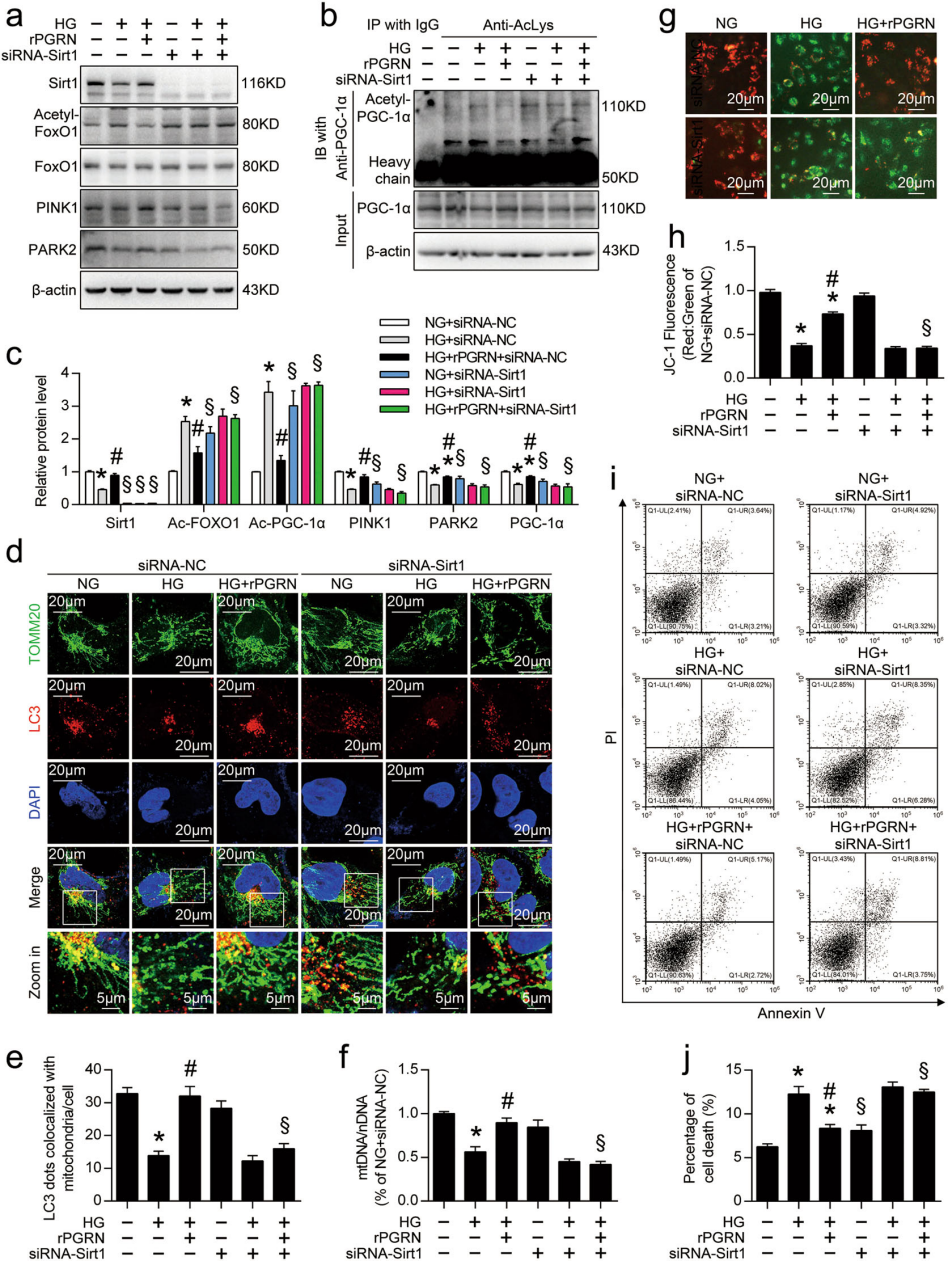
Inhibition of Sirt1 expression counteracted the protective effects of PGRN in podocytes with HG treatment. **a** Representative western blot gel documents of acetylated-FoxO1, FoxO1, PINK1, and PARK2 in podocytes with different treatments. **b** Representative western blot gel documents of acetylated-PGC-1α and PGC-1α in podocytes with different treatments. **c** Summarized data showing the levels of acetylated-FoxO1 and acetylated-PGC-1α relative to FoxO1 and PGC-1α, and the expression levels of PINK1, PARK2, and PGC-1α in podocytes with different treatments. **d** Representative confocal microscopic images of double immunofluorescence staining for TOMM20 (green) and LC3 (red) in podocytes with different treatments. Nuclei were revealed using DAPI staining. **e** Quantitative analysis of mitophagosome formation represented by colocalization of autophagosomes (LC3) and mitochondria (TOMM20). **f** Relative mitochondrial DNA content (mtDNA:nDNA) in podocytes with different treatments. The results were normalized to the ratio of mtDNA:nDNA of NG-treated podocytes with siRNA-NC transfection. **g** Representative images of podocytes stained with JC-1 in live cultured podocytes with different treatments. **h** Quantification of JC-1 fluorescence (red-to-green ratio) showing changes in fluorescence intensity in podocytes with different treatments. JC-1 fluorescence was normalized with the red-to-green ratio of NG-treated podocytes with siRNA-NC transfection. **i** Representative flow cytometry analysis depicting the detection of apoptosis in podocytes with different treatments stained with fluorescein isothiocyanate (FITC)-conjugated Annexin V and propidium iodide (PI). **j** Quantitative data expressing overall percentage of cell death (annexin V-FITC-positive), including the amount of apoptotic and necrotic cells determined by flow cytometric analysis in podocytes with different treatments. *P < 0.05 vs. NG treatment, ^#^*P* < 0.05 vs. HG treatment, ^§^*P* < 0.05 vs. siRNA-NC

As shown in Fig. 7a–c, gene silencing of Sirt1 markedly restricted PGRN-reduced acetylation of FoxO1 and PGC-1α in podocytes under HG condition. Western blot analyses also showed that inhibition of Sirt1 expression diminished PGRN-enhanced levels of PINK1, PARK2, and PGC-1α in HG-treated podocytes (Fig. 7a–c). Functionally, inhibition of Sirt1 expression in HG-treated podocytes also disturbed PGRN-restored formation of mitophagosomes (Fig. 7d, e), mtDNA:nDNA (Fig. 7f). Finally, inhibition of Sirt1 expression counteracted the protective effects of PGRN on mitochondrial function (Fig. 7g, h), and cell fates (Fig. 7i, j) of podocytes with HG treatment.

### Administration of rPGRN protected against podocyte injury in mice with DN

**Fig. 8:**
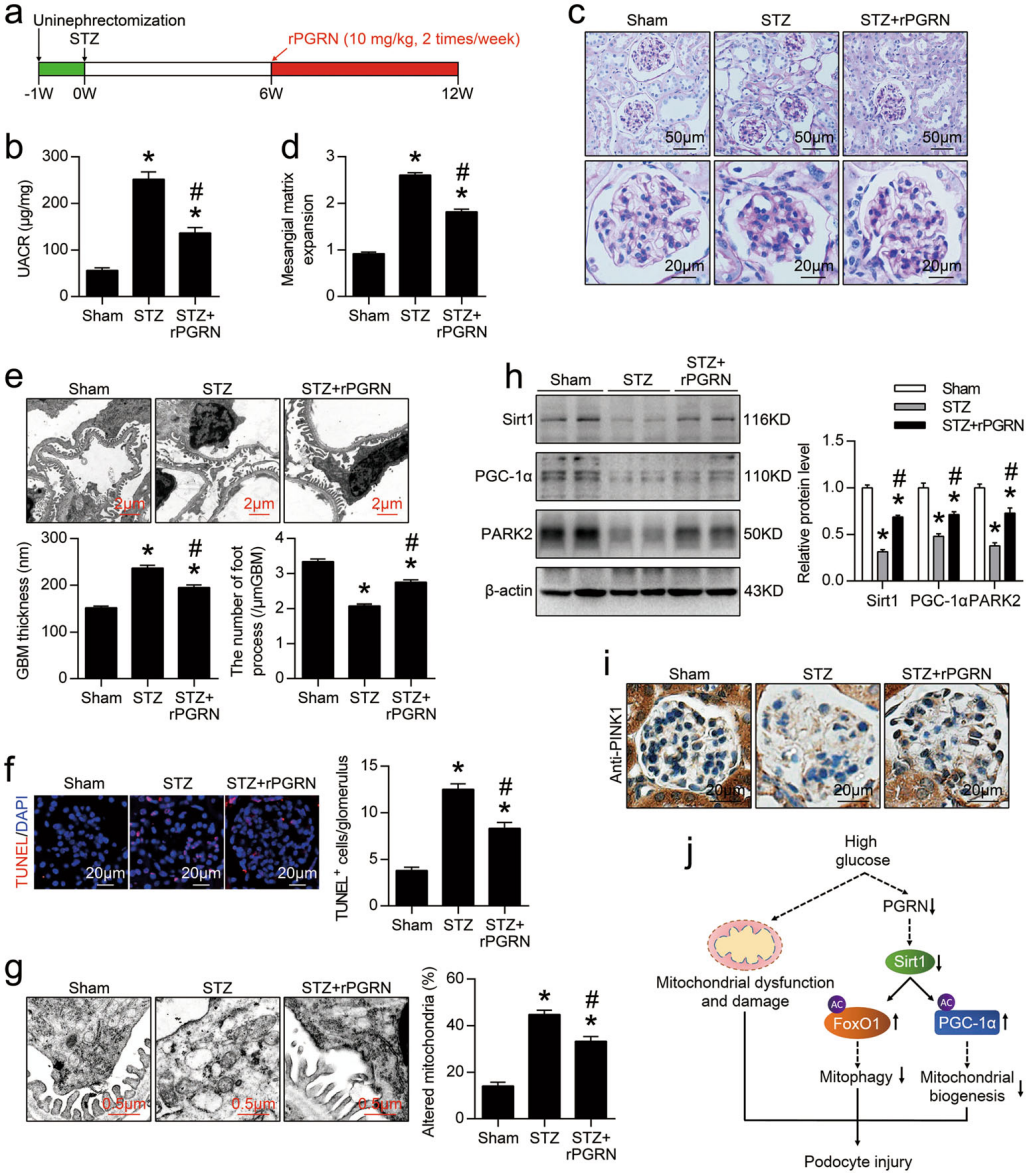
Administration of recombinant human PGRN (rPGRN) protected against podocyte injury in mice with DN. **a** A representative figure showing the procedure of rPGRN treatment in this study. **b** Urine albumin-to-creatinine ratio (UACR) in different groups of mice. **c** Representative images of PAS staining of kidney sections in different groups of mice. **d** Quantification of Mesangial Matrix Index of glomerulus from different groups of mice. **e** Representative TEM images showing morphological changes in the podocyte foot process in different groups of mice. Quantitative assessment of glomerular filtration barrier integrity, including GBM thickness and the number of foot processes/μm GBM. **f** In situ TUNEL assays were performed to assess the cell death in glomerulus from different groups of mice. Nuclei were revealed using DAPI staining. Quantitative assessment of the number of cell death (number of TUNEL-positive cells per glomerulus). **g** Representative TEM images of glomeruli demonstrating mitochondrial morphology in podocytes of kidney sections. Quantification of the percentage of altered mitochondria in podocytes from different groups of mice. **h** Representative western blot gel documents and summarized data showing the expression levels of Sirt1, PGC-1α, and PINK2 in kidney from different groups of mice. *P < 0.05 vs. sham-operated mice, ^#^*P* < 0.05 vs. WT diabetic mice (*n* = 6). **i** Representative photomicrographs of PINK1 immunohistochemical staining in the kidney from different groups of mice. **j** Schematic representation showing that PGRN plays an important role in maintaining mitochondrial homeostasis to prevent podocyte injury in diabetic nephropathy by regulating Sirt1-PGC-1α/FoxO1 signaling-mediated mitochondrial biogenesis and mitophagy

To examine the therapeutic potential of PGRN in DN, rPGRN was administrated to STZ-induced diabetic mice by intraperitoneal injection twice per week beginning at 6 weeks following STZ injection (Fig. 8a), rPGRN administration for 6 weeks significantly ameliorated renal injury as evidenced by reduced the levels of urinary albumin excretion (Fig. 8b), decreased mesangial expansion (Fig. 8c, d), and ameliorated podocyte injury (Fig. 8e) in DN mice, as well as decreased apoptosis in glomeruli (Fig. 8f). In addition, mitochondrial abnormalities in podocytes and glomerular endothelial cells of diabetic mice were alleviated by rPGRN administration (Fig. 8g, Supplementary Fig. 2b). Consistently, the levels of Sirt1, PGC-1α, PARK2 in kidney (Fig.8h) and PINK1 in glomeruli (Fig.8i) were significantly increased after rPGRN administration in diabetic mice.

## Discussion

Mitochondrial dysfunction is considered as a key mediator in the pathogenesis of DN and other glomerulopathy^24,25^ due to the influence in cellular metabolism. Therefore, therapeutic strategies to address mitochondrial dysfunction hold considerable promise for the treatment of DN. In this study, we observed that the levels of PGRN were significantly reduced in the kidney from diabetic mice and patients with biopsy-proven DN compared with healthy controls. We further found that under the normal conditions, PGRN deficiency in mice had no effects on renal function including the podocyte structure and function, which was consistent with previous studies showing that PGRN knockout mice display normal life expectancy with behavioral deficits and progressive neuropathology but few other recorded phenotypes^26^. Although a recent study has shown that PGRN deficiency mice develop progressive polydipsia and polyuria due to increased drinking behavior and urinary concentrating defect during aging (starting at 6 months), the renal function of aged *Grn*^*−/−*^ mice were in the normal range and there were no overt signs of a glomerular or tubular pathology^27^. In this study, we found that there was no significant difference in mitochondrial morphology of podocytes, kidney morphology and urinary albumin excretion between *Grn*^*−/−*^ mice (<5 months) and age-matched WT mice under normal condition. However, under diabetic conditions, PGRN deficiency exacerbated podocyte injury and proteinuria. Meanwhile, considering that PGRN deficiency or administration of rPGRN (Supplementary Table 1, Supplementary Fig. 3) had no effect on the blood glucose levels in diabetic mice, we concluded that the effects of PGRN in diabetic mice are likely specific to the kidney, not because of the regulation of carbohydrate metabolism.

One of the most important findings was the role of PGRN in maintaining mitochondrial homeostasis by the regulation of mitochondrial biogenesis and mitophagy. PPAR-γ coactivator-1α (PGC-1α) is a transcriptional coactivator that has been identified as a prominent regulator of mitochondrial biogenesis. Previous studies have found that the level of PGC-1α was reduced in kidney biopsies from patients with DN^6^ and defects in mitochondrial biogenesis with reduced expression or activity of PGC-1α was recognized to contribute to DN^28^. Consistently, we also found that PGC-1α and associated mitochondrial gene levels were suppressed in podocytes under HG condition. Upon induction of PGC-1α by rPGRN treatment, mitochondrial DNA content and respiratory chain function were restored accompanied by inducing the expression of molecules involved in mitochondrial DNA replication/translation, such as TFAM, indicating that PGRN may promote recovery of podocyte injury, at least in part, by targeting PGC-1α and mitochondrial biogenesis.

On the other hand, we also find that PGRN positively regulates mitophagy, which is one of the selective autophagy processes to remove accumulated damaged or dysfunctional mitochondria from cells to maintain mitochondrial homeostasis^29^. Mitophagy is regulated by a PINK1/PARK2 mechanism that tags mitochondria for degradation^30^. Alteration in PINK1 and PARK2 expression was observed in tubular cells and podocytes in experimental models of DN^31^. In this study, the decreased-expression of PINK1 in HG-treated podocytes and glomeruli from diabetic mice was restored by rPGRN treatment both in vitro and in vivo. Furthermore, rPGRN treatment enhanced mitophagy and ameliorated podocyte injury. Collectively, our results elucidate the novel role of PGRN in maintaining mitochondrial homeostasis in podocytes, by selective elimination of dysfunctional mitochondria through mitophagy and induction of mitochondrial biogenesis in DN.

Mechanistically, we find that Sirt1 plays a pivotal role linking PGRN to mitochondrial homeostasis. Sirt1, a member of the sirtuin family, is a nicotinamide adenine dinucleotide (NAD^+^)-dependent protein deacetylase and master metabolic regulator. Sirt1 is also involved in a variety of mitochondrial processes, including the electron transport chain, tricarboxylic acid cycle, fatty acid oxidation, redox homeostasis, and mitochondrial biogenesis^32^. Sirt1 can stimulate mitochondrial biogenesis via the PGC-1α pathway in an AMPK-independent manner, thereby enhancing mitochondrial recovery^33^. Recent studies highlight the importance of sirtuins on the regulation of renal function^16,34^. We recently assessed the expression patterns of sirtuins in the kidney from STZ-induced diabetic mice showing that the levels of Sirt1, Sirt3, Sirt4, and Sirt6 were reduced in the kidney from STZ-induced diabetic mice^16^. In this study, we further found that only the expression of Sirt1 was markedly restored in podocytes under HG condition by rPGRN treatment, indicating that PGRN specifically mediates Sirt1 expression. In podocytes, Sirt1 has been demonstrated to regulate the expression of target genes, which act to maintain podocyte function by modulating the levels of histone acetylation^35,36^. Sirt1 can regulate PGC-1α activity and energy metabolism of mitochondria by its deacetylation^25,37^. In the kidney, the activation of the Sirt1-PGC-1α signaling in mitochondria ameliorates aldosterone-induced podocyte injury^25^. Consistently, we found that PGRN rescued HG-reduced Sirt1 expression, thereby decreasing PGC-1α acetylation, resulting in the activation of PGC-1α and enhancing mitochondria biogenesis. Besides reducing acetylation of PGC-1α, we also found that PGRN-Sirt1 signaling regulates the activity of transcription factor FoxO1, which plays an important role in aging, cell metabolism, insulin resistance, and oxidative stress resistance. FoxO1 phosphorylation by PI3K/Akt leads to the export of FoxO1 from the nucleus to the cytoplasm, thereby inhibiting its transcription activity. FoxO1 activity is also regulated by acetylation on specific lysine residues. CREB-binding protein acetylates FoxO1 and disrupts FoxO1-DNA interactions, thereby attenuating FoxO1-dependent gene expression. Conversely, Sirt1 increases FoxO1 DNA-binding ability by deacetylating FoxO1 and potentiates its transcription activity. Thus, FoxO1 and Sirt1 act synergistically to modulate diverse cellular processes. Of note, FoxO1 promotes mitophagy via PINK1/PARK2 pathway to protect against mitochondrial dysfunction and podocyte injury under HG condition^10^. In this study, we found that PGRN decreased the acetylation of FoxO1 by Sirt1 in HG-treated podocytes. Gene silencing of Sirt1 attenuated PGRN-induced expression of PINK1 and PARK2, mitophagy, and survival of podocytes under HG condition, indicating that Sirt1-FoxO1 signaling confers mitochondrial protection by mediating mitophagy. Despite currently the mitochondria-targeting therapeutics as effective interventions to preserve mitochondria structures and functions have been demonstrated in several animal models of renal injuries, and some therapeutic interventions are now under consideration for clinical trials such as MitoQ and SS-31, the delivery of specific therapies for DN is still a great challenge. Finally, we provided direct evidence for therapeutic potential of PGRN in mice with DN by administration of rPGRN, expecting to translate these discoveries into therapeutic strategies to ameliorate renal injuries in DN in the future.

Although in this study, we did not explore how secreted PGRN acts on podocytes, some studies have demonstrated that PGRN can bind to and activate a number of receptors, such as Ephrin A2^38^ and TNF receptors^12^. In addition, intracellular PGRN is also localized to vesicular structures and promotes vesicle transport and vesicular transmembrane transport of ions and proteins^39^. Sortilin is a multiligand receptor that traffics protein from the Golgi to the endosomes, secretory vesicles, and the cell surface. Sortilin was reported to bind with PGRN, forming an endocytosis-mediating complex, and to mediate the delivery of PGRN to the endosome/lysosomal pathway in neurons^40^. In particular, sortilin was also expressed in both intracellular and surface of podocytes^41^. Therefore, it is possible that PGRN binds to its membrane receptors such as Ephrin A2 and TNF receptors or be internalized by sortilin-mediated endocytosis to put efforts on specific cascades, thereby activating Sirt1-PGC-1α/FoxO1 signaling. Further studies need to be confirmed this hypothesis.

In addition, it should be noted that except for podocytes, we also found PGRN deficiency exacerbated GEC injury and mesangial expansion in diabetic mice, suggesting that PGRN deficiency has detrimental effects on different glomerular cells individually under diabetic conditions or it may exist the potential implications of crosstalk among the cells in the glomerulus. In particular, numerous studies have shown the crosstalk among glomerular cells. Podocyte injury frequently results in mesangial cell proliferation, whereas mesangial cell injury leads to foot process fusion and proteinuria^42^. In DN, GEC injury leads to podocyte damage, while podocyte loss further exacerbates GEC injury^43^. Thus, a complex crosstalk among glomerular cells appears to contribute to the pathogenic mechanism of DN. Mechanistically, vascular endothelial growth factor (VEGF) may be one of the major mediators for the communication among podocytes, GECs and mesangial cells. Podocytes act as an important source of glomerular VEGF^44^. VEGF-A produced by podocytes is required for differentiation and survival of GECs and mesangial cells^45,46^. Recent studies have shown that PGRN promotes the expression of VEGF-A in colorectal cancer cells^47^. Therefore, the GECs injury and mesangial expansion in PGRN-deficient diabetic mice may be due to the reduced VEGF-A production in PGRN-deficient podocytes. Of course, we cannot exclude that PGRN can directly acts as a mediator on the regulation of GECs and mesangial cells individually, relying on its ability to promote survival and angiogenesis, although our in vitro study found that PGRN was not sensitive to HG condition in glomerular endothelial cells and mesangial cells. In fact, the podocyte-specific PGRN knockout mouse would be an ideal model for investigating podocyte PGRN function and clarify this issue. These findings need to be further confirmed once the podocyte-specific PGRN knockout mice are available.

Collectively, our studies for the first time demonstrate that PGRN protects against podocyte injury in DN, at least in part through maintaining mitochondrial homeostasis via PGRN- Sirt1-PGC-1α/FoxO1 signaling-mediated mitochondrial biogenesis and mitophagy (Fig. 8j), suggesting that PGRN may be an innovative therapeutic strategy for treating patients with DN.

## Supplementary information

Revised Supplemental Material
