# Supplementary material for: PGRN acts as a novel regulator of mitochondrial homeostasis by facilitating mitophagy and mitochondrial biogenesis to prevent podocyte injury in diabetic nephropathy
Source: Cell Death Dis. 2019 Jul 8;10(7):524. doi: 10.1038/s41419-019-1754-3 (PMC6614416; doi:10.1038/s41419-019-1754-3)
Supplement: Supplementary file 1 — Revised Supplemental Material [file 41419_2019_1754_MOESM1_ESM.docx]

**Supplemental Material**

**PGRN acts as a novel regulator of mitochondrial homeostasis by facilitating mitophagy and mitochondrial biogenesis to prevent podocyte injury in diabetic nephropathy**

Di Zhou^1^, Meng Zhou^1^, Ziying Wang^1^, Yi Fu^1^, Meng Jia^1^, Xiaojie Wang^1^, Min Liu^1^, Yan Zhang^1^, Yu Sun^1^, Yi Lu^3^, Wei Tang^2*^, and Fan Yi^1,4*^

^1^The Key Laboratory of Infection and Immunity of Shandong Province, Departments of Pharmacology, School of Basic Medical Sciences, Shandong University, Jinan, China

^2^Departments of Pathogenic Biology, School of Basic Medical Sciences, Shandong University, Jinan, China

^3^Departments of Biochemistry and Molecular Biology, School of Basic Medical Sciences, Shandong University, Jinan, China

^4^The State Key Laboratory of Microbial Technology, Shandong University, Jinan, China

**Supplementary Table 1. Physical and biochemical parameters of experimental animals.**

| **Variable** | | **Wild-type mice** | | ***Grn^-/-^* mice** | |
| --- | --- | --- | --- | --- | --- |
|  |  | **Sham** | **STZ** | **Sham** | **STZ** |
| **Body weight (g)** | | 24.61±0.87 | 21.54±1.49 | 24.19±0.67 | 22.37±1.52 |
| **Heart rate (beat/min)** | | 524.2±23.79 | 525.2±14.58 | 525.0±23.62 | 534.2±25.08 |
| **Blood pressure (mmHg)** | **Systolic** | 111.3±1.73 | 113.2±1.87 | 110.7±4.23 | 113.7±1.54 |
|  | **Diastolic** | 70.33±3.23 | 70.17±2.023 | 70.83±2.65 | 70.50±2.14 |
| **FBG (mM)** | | 5.87±0.42 | 21.53±1.89* | 5.95±0.53 | 23.43±1.85* |
| **Relative kidney weight / body weight (mg/g)** | | 9.25±0.56 | 14.25±0.62* | 10.27±0.90 | 17.76±0.96*# |

Male mice were uninephrectomized. After a 1-week recovery period from uninephrectomy, diabetes was induced by intraperitoneal injection of streptozotocin (50 mg/kg body weight for 4 consecutive days). All mice had unrestricted access to food/water and were maintained for 12 weeks. FBG, fasting blood glucose. Values are means ± S.E.M. for 6 mice in each group. **P*<0.05 vs. sham-operated mice; #*P*<0.05 vs. STZ-induced diabetic wild-type mice.

**Supplementary Table 2. Primer pairs used in this study.**

| **Primer pairs for real time RT-PCR** | | | | |
| --- | --- | --- | --- | --- |
| **Genes** | **Accession No.** | **Forward** | | **Reverse** |
| Mus PGRN | NM_008175.4 | GGTTGATGGTTCGTGGGGATGTTG | | AAGGCAAAGACACTGCCCTGTTGG |
| Mus β-actin | NM_007393.5 | GGCTGTATTCCCCTCCATCG | | CCAGTTGGTAACAATGCCATGT |
| Homo PINK1 | NM_032409.2 | TGGAGGTGACAAAGAGCACC | | AACCTGCCGAGATGTTCCAC |
| Homo PGC-1α | NM_001354828.1 | TCTGAGTCTGTATGGAGTGACAT | | CCAAGTCGTTCACATCTAGTTCA |
| Homo TFAM | NM_001270782.1 | CGCTCCCCCTTCAGTTTTGT | | CCAACGCTGGGCAATTCTTC |
| Homo PARK2 | NM_013987.2 | CACCAGCATCTTCCAGCTCA | | TCGCCTCCAGTTGCATTCAT |
| Homo β-actin | NM_001101.4 | GAAGTGTGACGTGGACATCC | | CCGATCCACACGGAGTACTT |
| **Primer pairs for quantification of mitochondrial DNA content** | | | | |
| **Genes** | **Forward** | | **Reverse** | |
| Homo Cytb | TCACCAGACGCCTCAACCGC | | GCCTCGCCCGATGTGTAGGA | |
| Homo COII | GGCACATGCAGCGCAAGTAGG | | GGCGGGCAGGATAGTTCAGACG | |
| Homo β-actin | AGCGGGAAATCGTGCGTGAC | | CGGACTCGTCATACTCCTGCT | |

**Supplementary Table 3. Antibodies used in this study.**

| Primary antibodies | Host | Dilution and supplier | Application |
| --- | --- | --- | --- |
| PGRN | Rabbit | 1:1000 for WB, 1:100 for IF and IHC; Santa Cruz, Dallas, TX | WB, IF, IHC |
| Synaptopodin | Goat | 1:100; Santa Cruz, Dallas, TX | IF |
| Nephrin | Rabbit | 1:1000 for WB, 1:50 for IF; Boster, Wuhan, China | WB, IF |
| Podocin | Rabbit | 1:1000 for WB, 1:50 for IF; Boster, Wuhan, China | WB, IF |
| PARP1 | Rabbit | 1:1000; Protein Tech Group, Chicago, IL | WB |
| Cleaved-caspase3 | Rabbit | 1:500 for WB, 1:100 for IF; Cell Signaling, Dancers, MA | WB，IF |
| PGC-1α | Mouse | 1:1000; Protein Tech Group, Chicago, IL | WB |
| TFAM | Rabbit | 1:1000; Protein Tech Group, Chicago, IL | WB |
| LC3B | Rabbit | 1:50; Cell Signaling, Danvers, MA | IF |
| PINK1 | Rabbit | 1:1000; Cell Signaling, Danvers, MA | WB |
| PINK1 | Rabbit | 1:50; Protein Tech Group, Chicago, IL | IHC |
| PARK2 | Mouse | 1:1000; Cell Signaling, Danvers, MA | WB |
| TOMM20 | Mouse | 1:100; BD Bioscience, San Jose, CA | IF |
| Sirt1 | Rabbit | 1:1000; Protein Tech Group, Chicago, IL | WB |
| Sirt2 | Rabbit | 1:1000; Protein Tech Group, Chicago, IL | WB |
| Sirt3 | Rabbit | 1:1000; Protein Tech Group, Chicago, IL | WB |
| Sirt4 | Rabbit | 1:1000; Protein Tech Group, Chicago, IL | WB |
| Sirt5 | Rabbit | 1:1000; Protein Tech Group, Chicago, IL | WB |
| Sirt6 | Rabbit | 1:1000; Protein Tech Group, Chicago, IL | WB |
| Sirt7 | Rabbit | 1:1000; Protein Tech Group, Chicago, IL | WB |
| FoxO1 | Rabbit | 1:1000; Cell Signaling, Danvers, MA | WB |
| Acetyl-FoxO1 | Rabbit | 1:1000; Santa Cruz, Dallas, TX | WB |
| Acetyl-lysine | Rabbit | 1:300; Cell Signaling, Danvers, MA | IP |
| β-actin | Mouse | 1:3000; Protein Tech Group, Chicago, IL | WB |
| COXIV | Rabbit | 1:150; Protein Tech Group, Chicago, IL | IHC |

**Supplementary Figure 1**

**
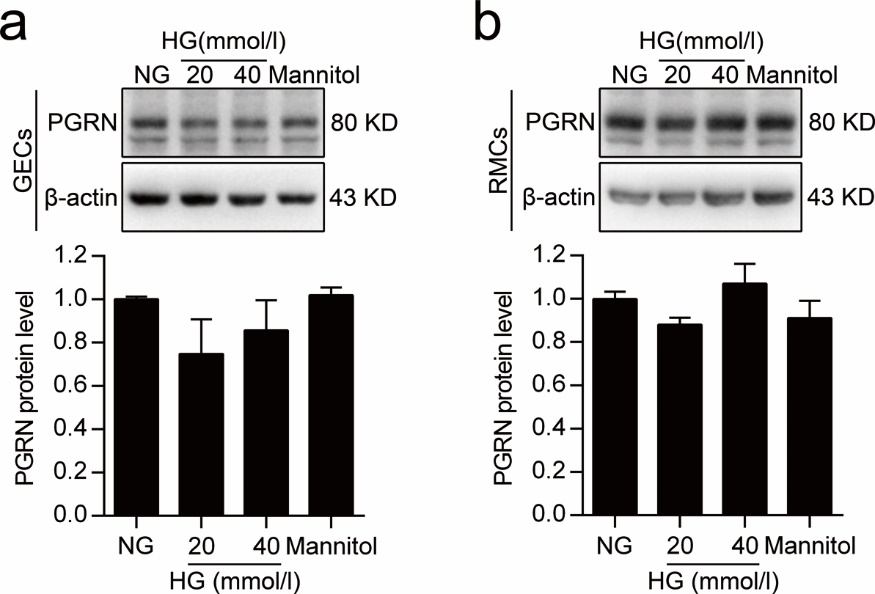
**

**Supplementary Figure 1.** Representative Western blot gel documents and summarized data showing the relative protein levels of PGRN in rat glomerular endothelial cells (GECs) **(a)** and rat mesangial cells (RMCs) **(b)** treated with high glucose (HG, final concentration 20 or 40 mmol/l in medium) for 48 h. *P<0.05 vs. NG treatment.

**Supplementary Figure 2**

**
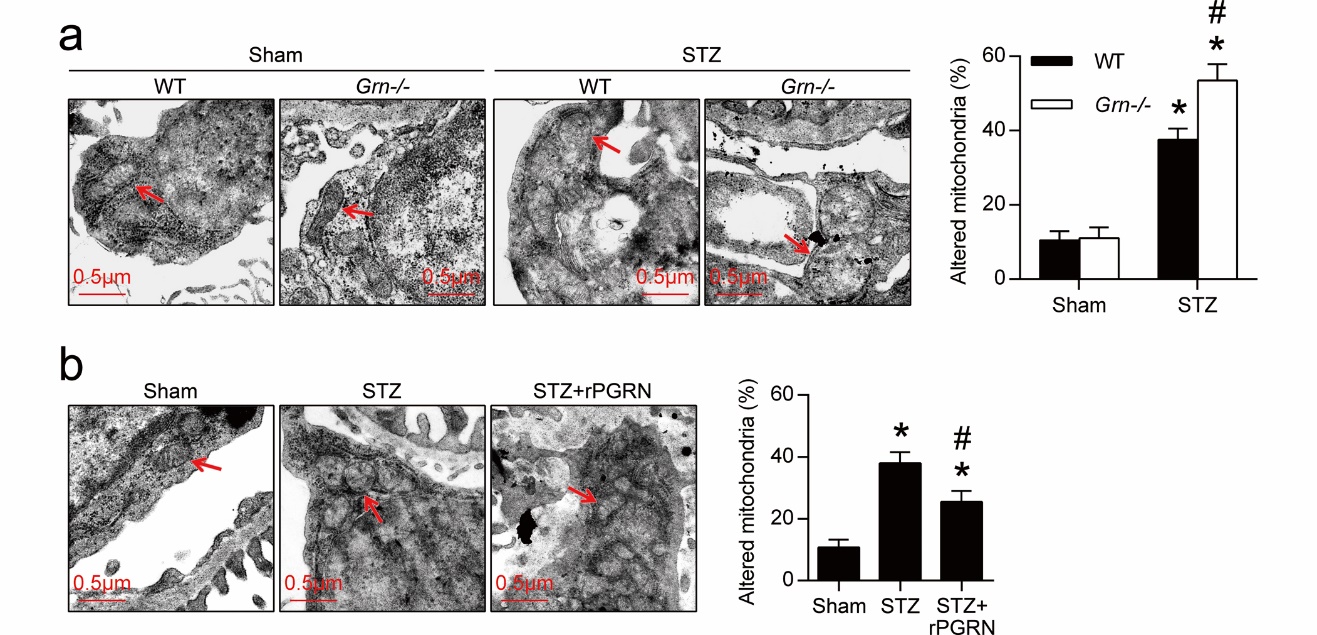
**

**Supplementary Figure 2.** PGRN deficiency exacerbated and rPGRN administration ameliorated mitochondrial damage in GECs from diabetic mice. **(a)** Representative TEM images of glomeruli demonstrating mitochondrial morphology in GECs of kidney sections. Quantification of the percentage of altered mitochondria in GECs from sham-operated and diabetic WT and *Grn^−/−^* mice. **(b)** Representative TEM images of glomeruli demonstrating mitochondrial morphology in GECs of kidney sections. Quantification of the percentage of altered mitochondria in GECs from sham-operated and diabetic WT mice, and rPGRN-administrated diabetic WT mice. *P < 0.05 vs. sham-operated mice, #P<0.05 vs. WT diabetic mice (n=6). Red arrows indicate representative mitochondria.

**Supplementary Figure 3**

**
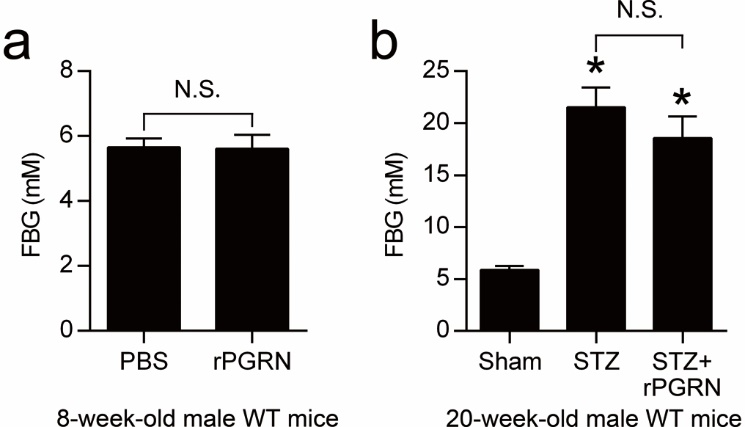
**

**Supplementary Figure 3.** rPGRN administration showed no effect on FBG levels of normal eight-week-old male WT mice and STZ-induced diabetic WT mice. **(a)** Fasting blood glucose (FBG) levels of eight-week-old male WT mice with PBS or rPGRN administration (n=8). N.S., not significant. **(b)** FBG levels of STZ-induced diabetic WT mice with or without rPGRN administration. *P < 0.05 vs. sham-operated mice (n=6), N.S., not significant.

**Supplementary Figure 4**

**
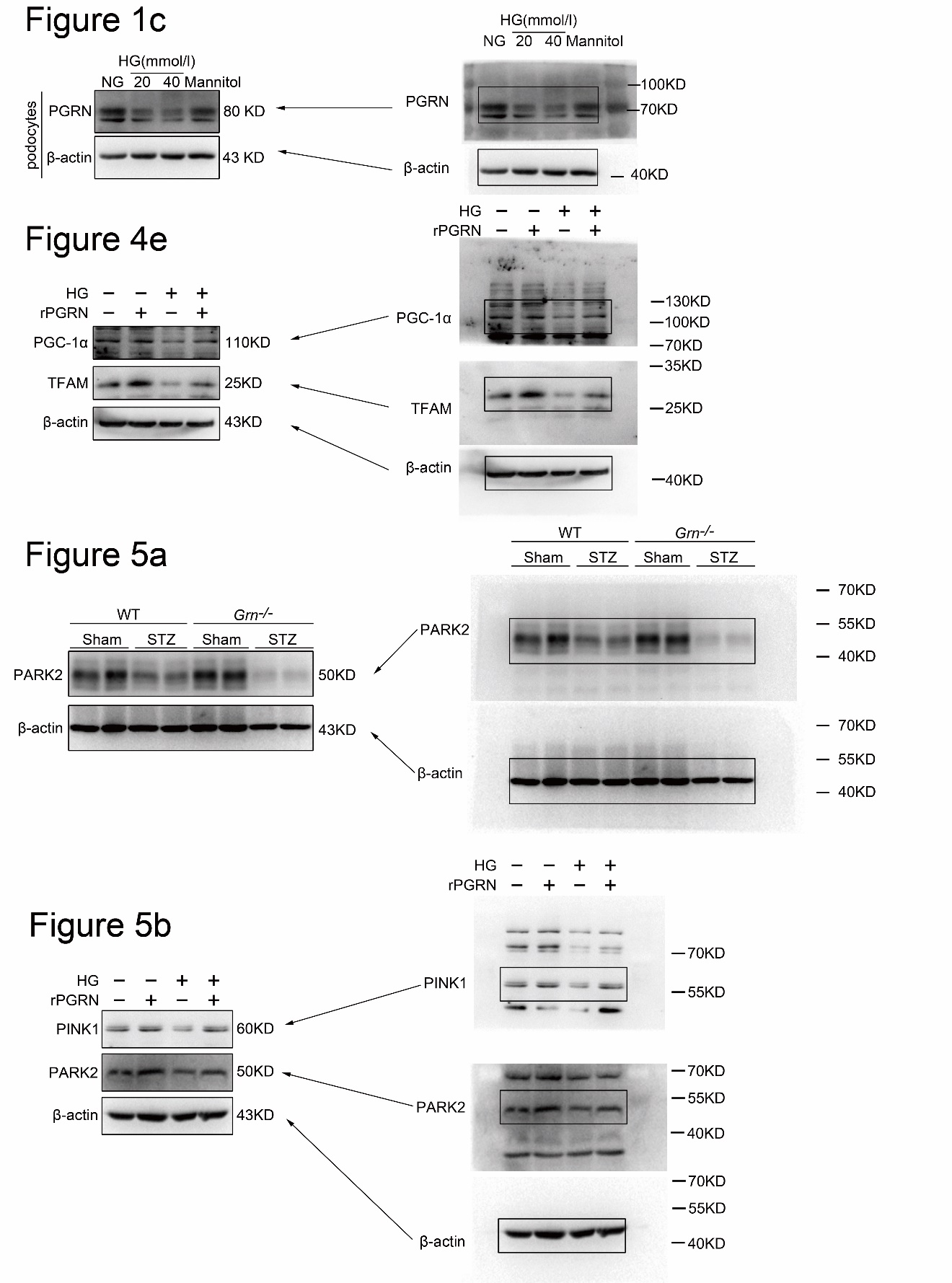
**

**
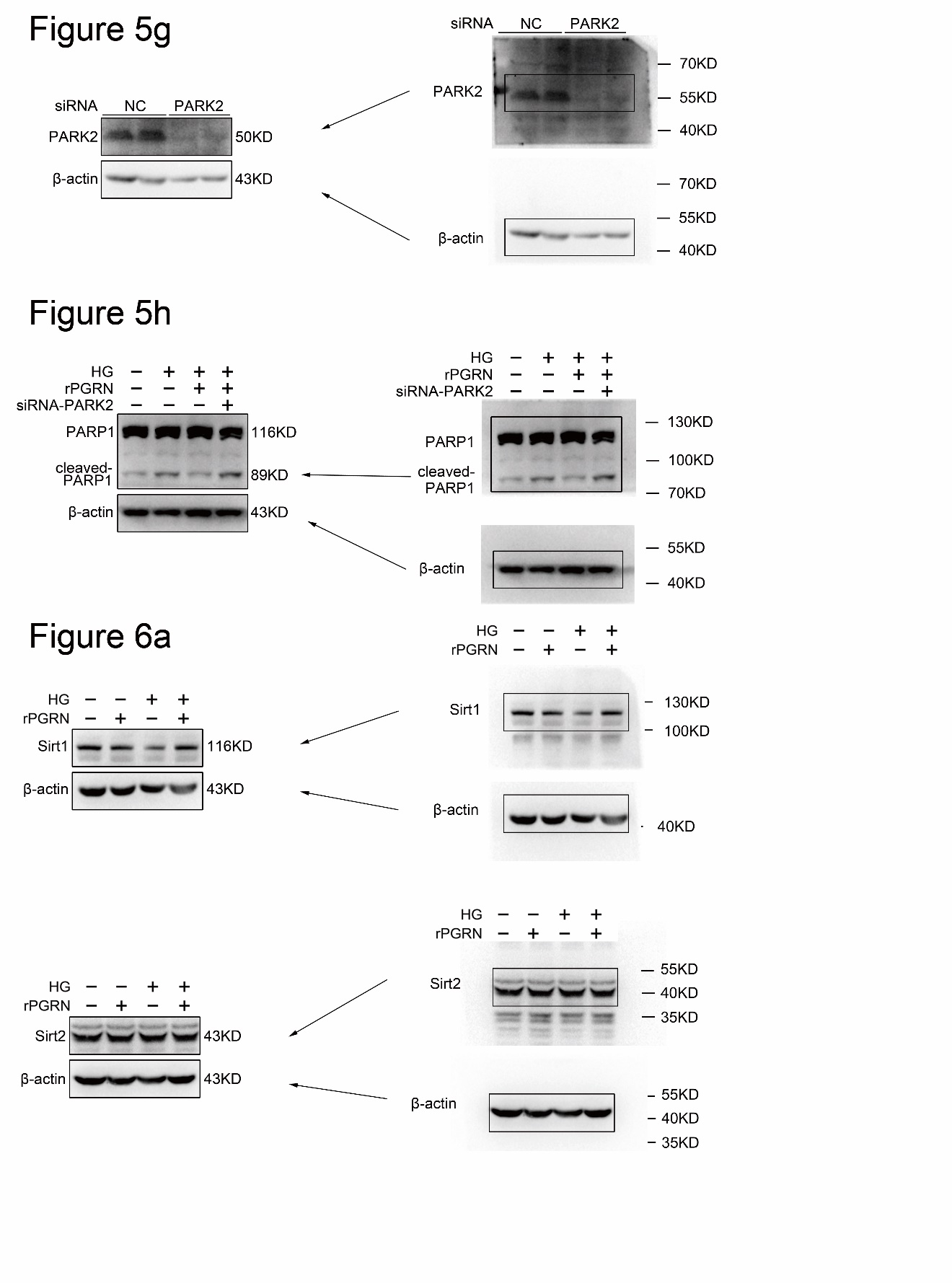
**

**
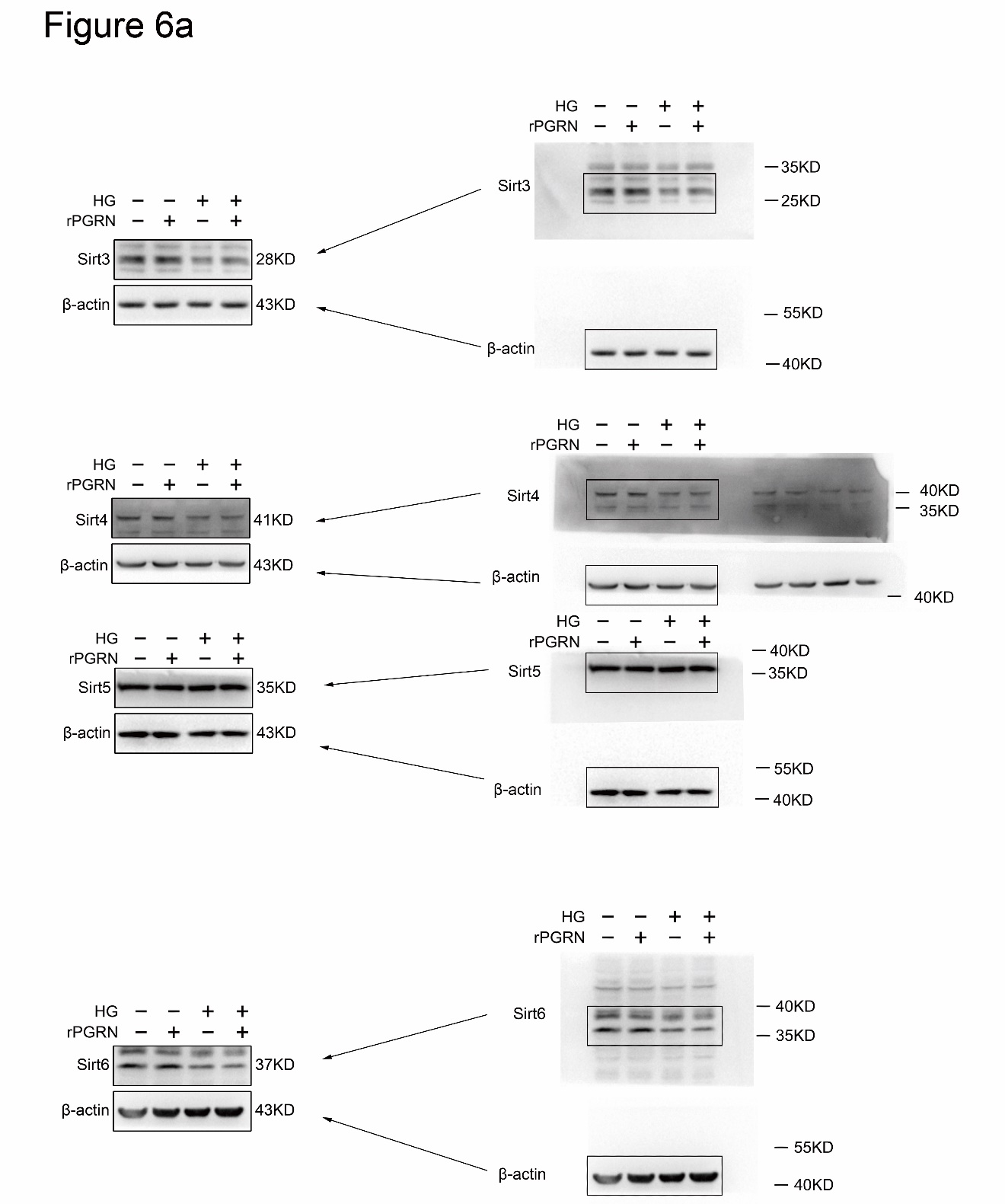
**

**
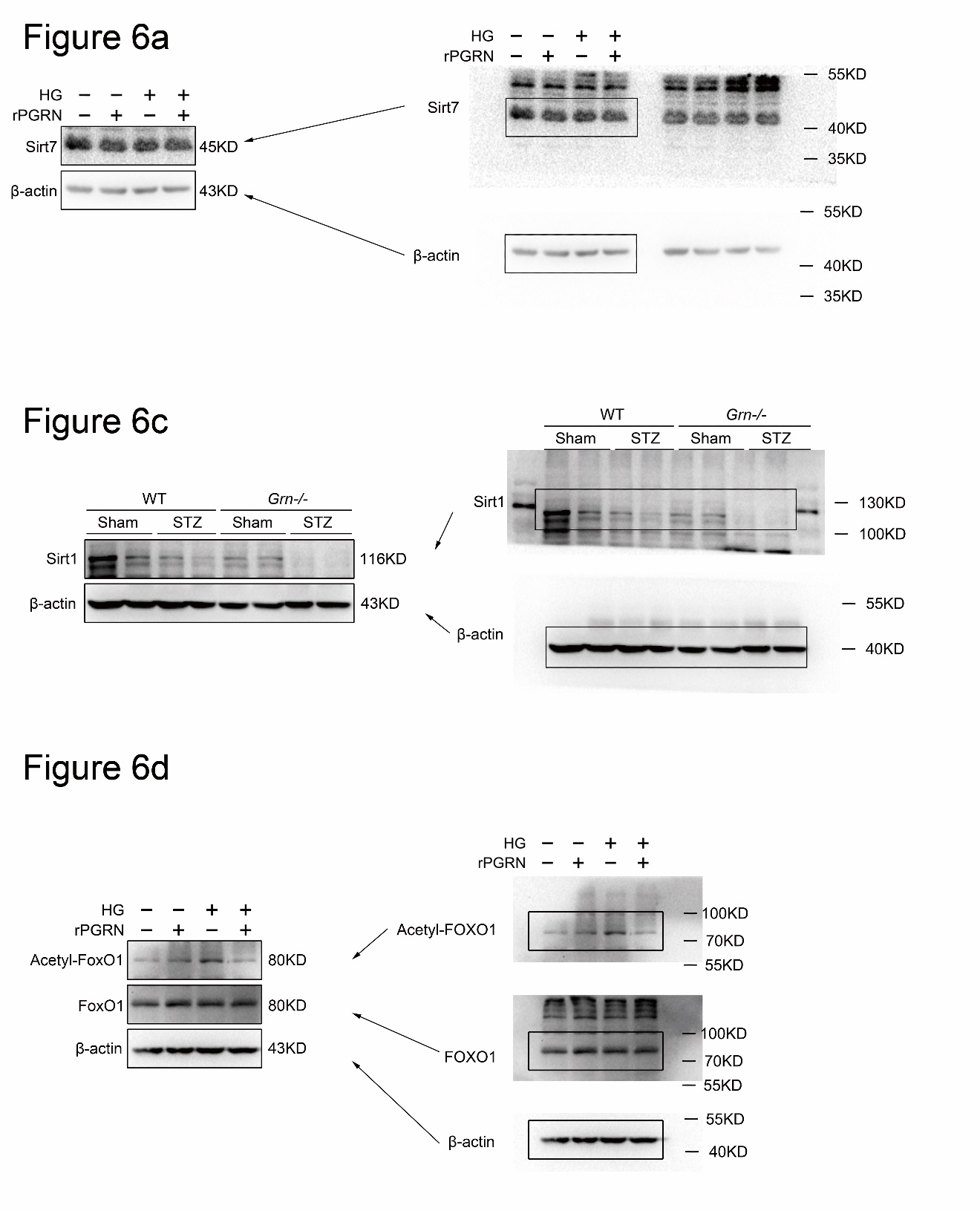
**

**
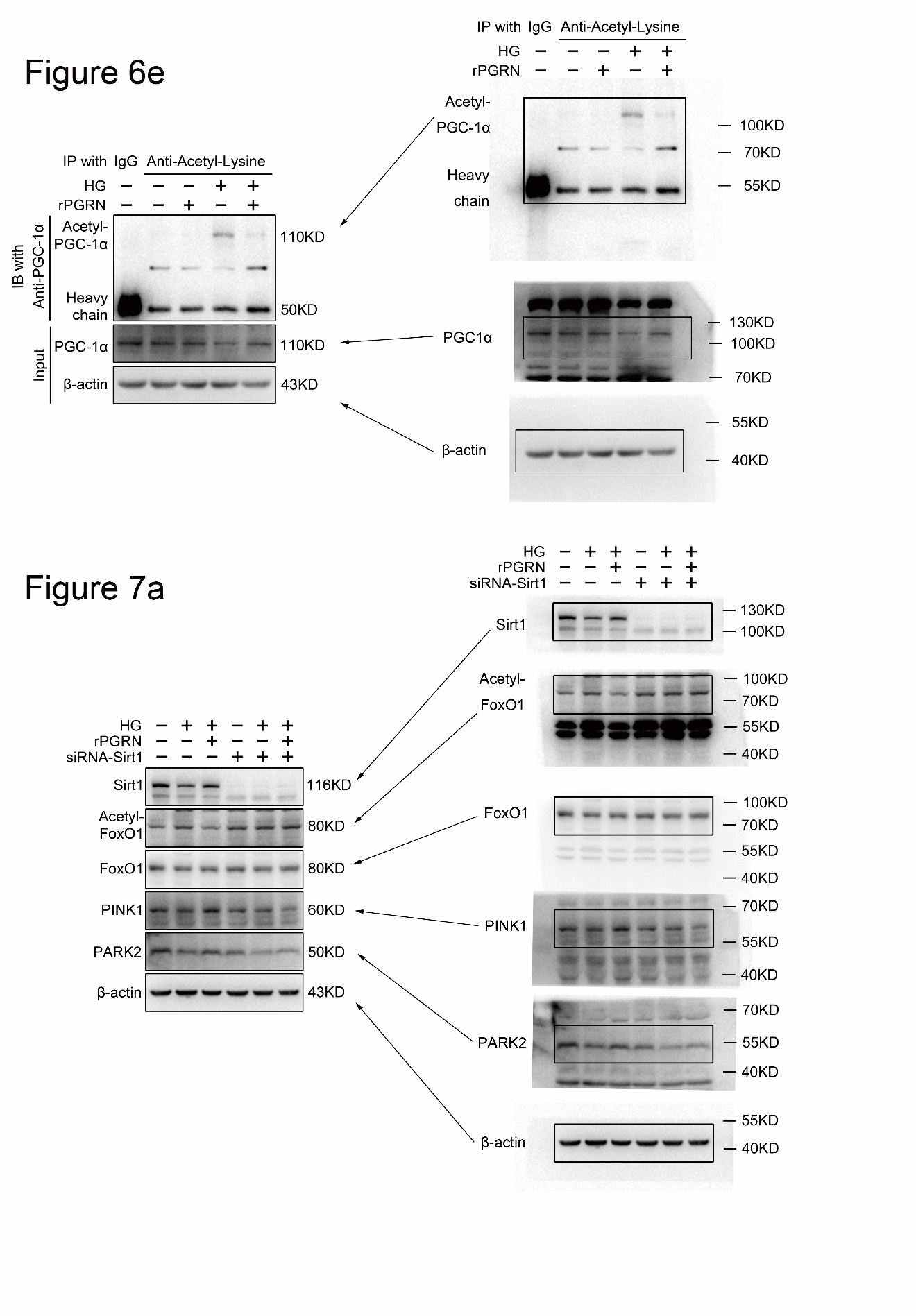
**

**
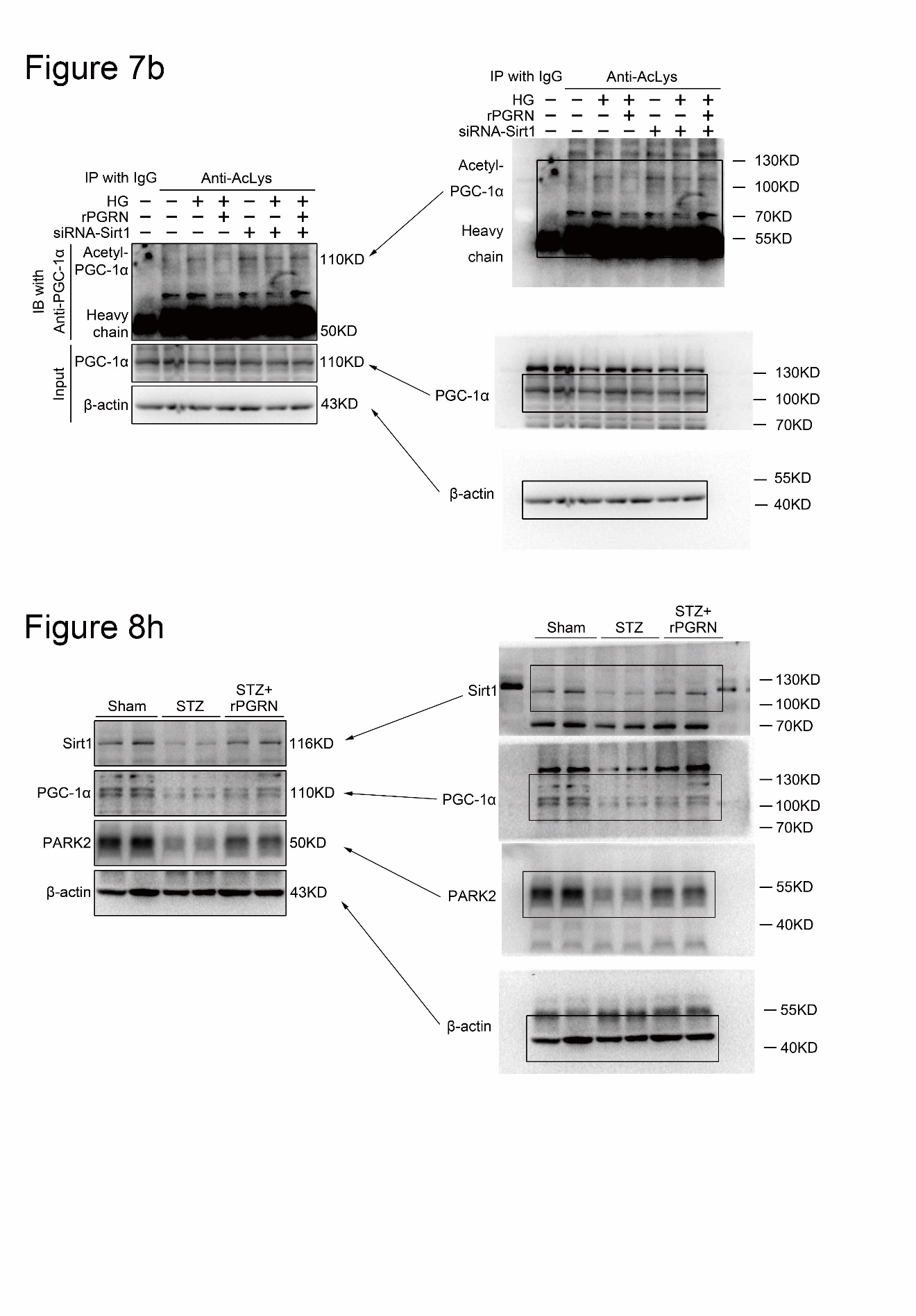
**

**
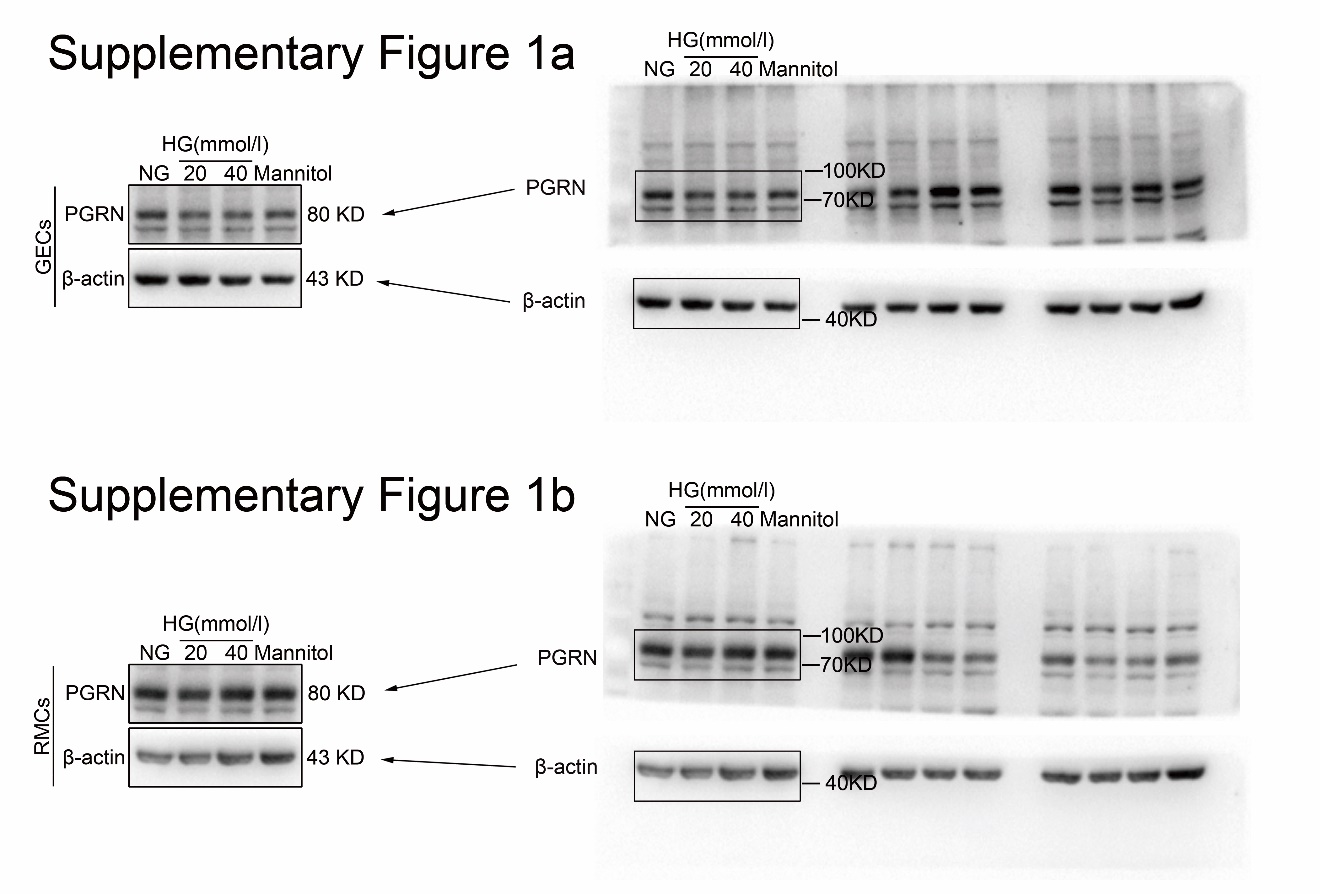
**

**Supplementary Figure 4.** Scans of the full films used to generate Western blot data for Figures 1-8 and Supplementary Figure 1.
